# Supplementary material for: Modeling tissue-specific breakpoint proximity of structural variations from whole-genomes to identify cancer drivers
Source: Nat Commun. 2022 Sep 26;13:5640. doi: 10.1038/s41467-022-32945-2 (PMC9512825; doi:10.1038/s41467-022-32945-2)
Supplement: Supplementary file 1 — Supplementary Information [file 41467_2022_32945_MOESM1_ESM.pdf]

Supplementary Figure 1

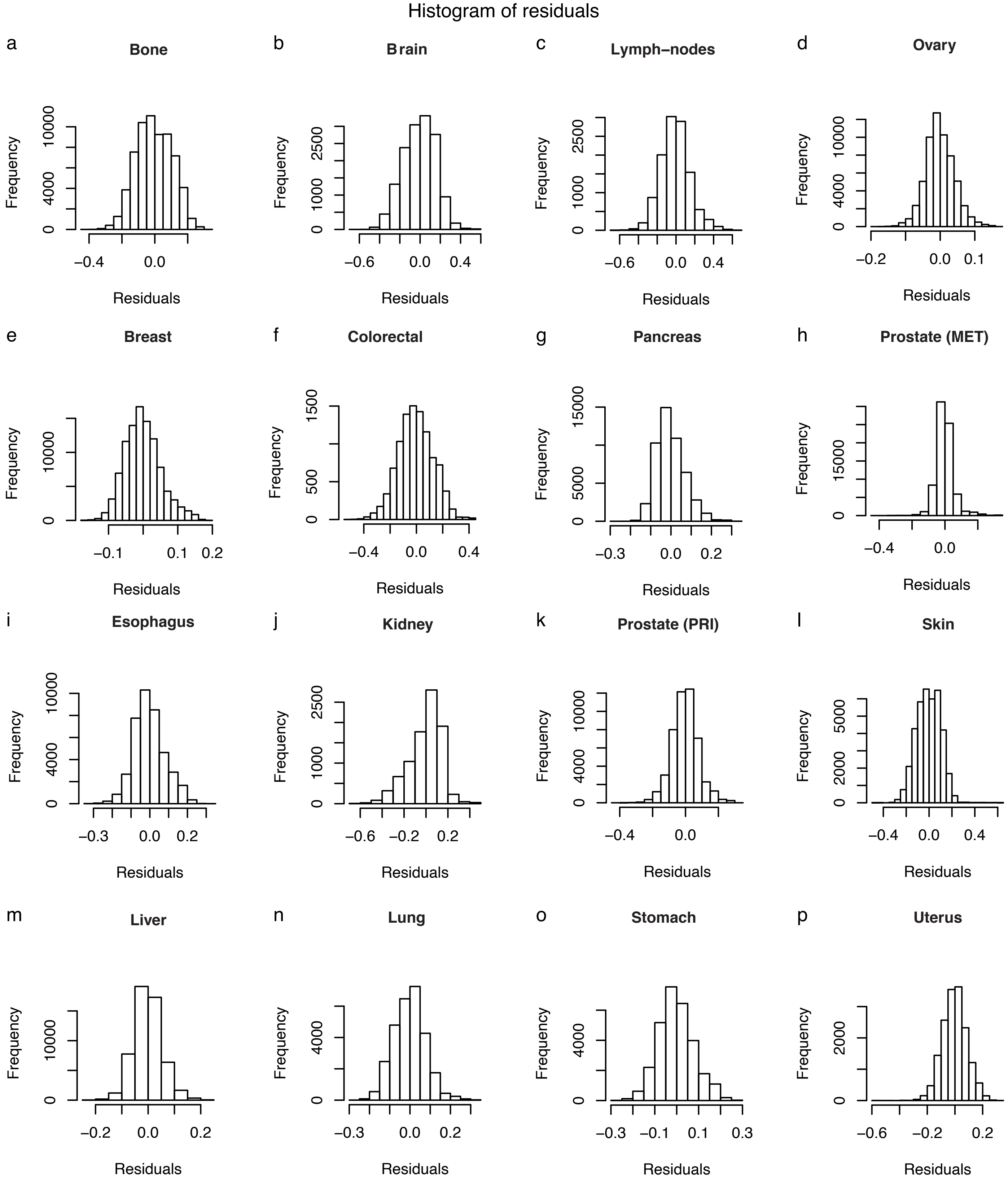

Histograms of the residuals obtained from GAM modeling.
Each panel (a-p), shows for each cancer cohort the plots of the GAM results. The plots show that for each cohort the prediction has narrow dispersion of residual around 0.

Supplementary Figure 2

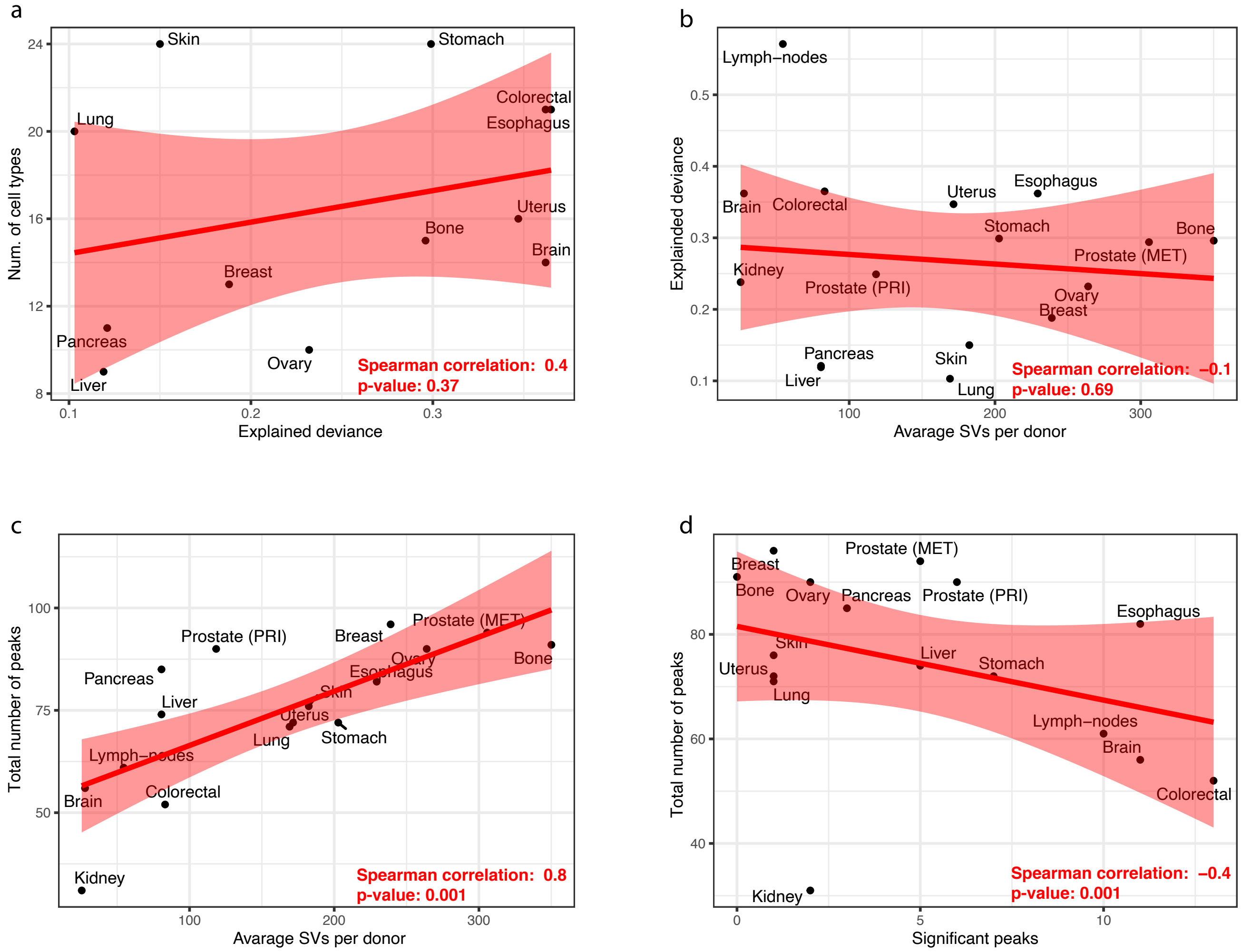

Correlation between GAM results and the cohort features.
(a) Positive Spearman correlation between cell type heterogeneity and explained deviance. (b) The explained deviance of the model does not correlate with the average number of SVs per sample in each cohort. (c) The total number of peaks in the BPpc of each cohort correlate with their average number of SVs per donor.
(d) Negative correlation between the total number of peaks and the number of significant rearranged peaks for each cohort. The red shade around the correlation line displays a 95% confidence interval.

Supplementary Figure 3a

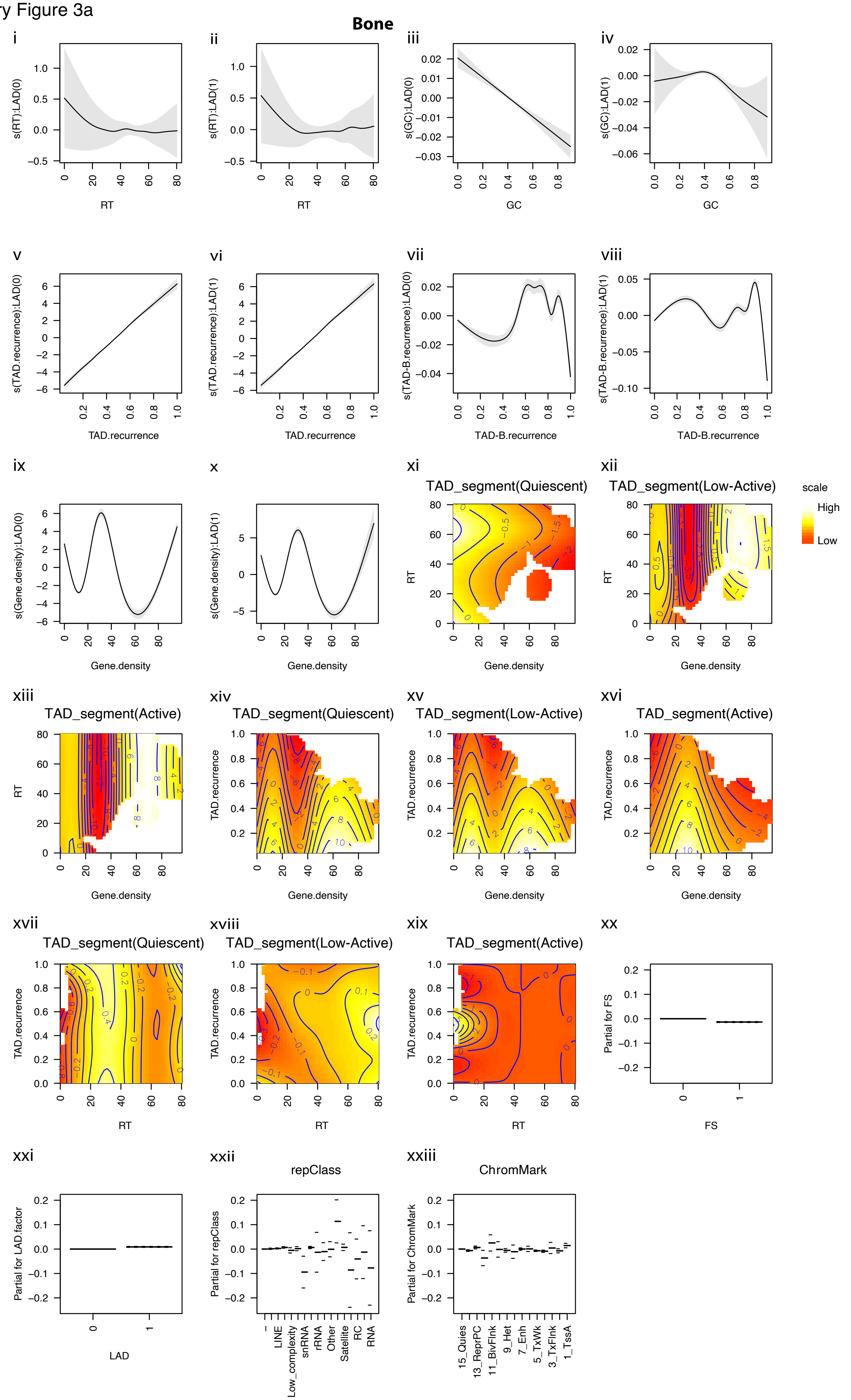

Supplementary Figure 3b

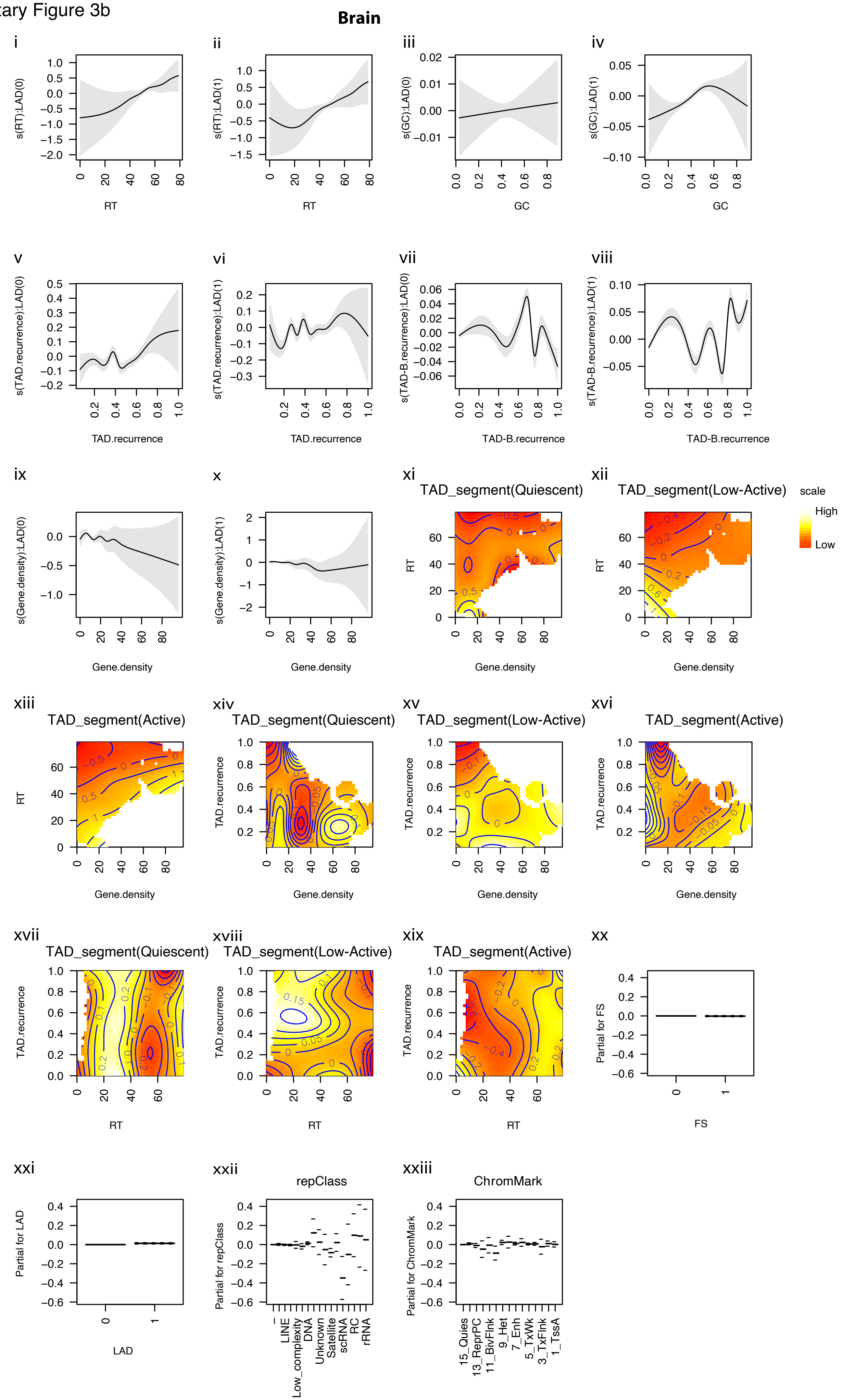

Supplementary Figure 3c

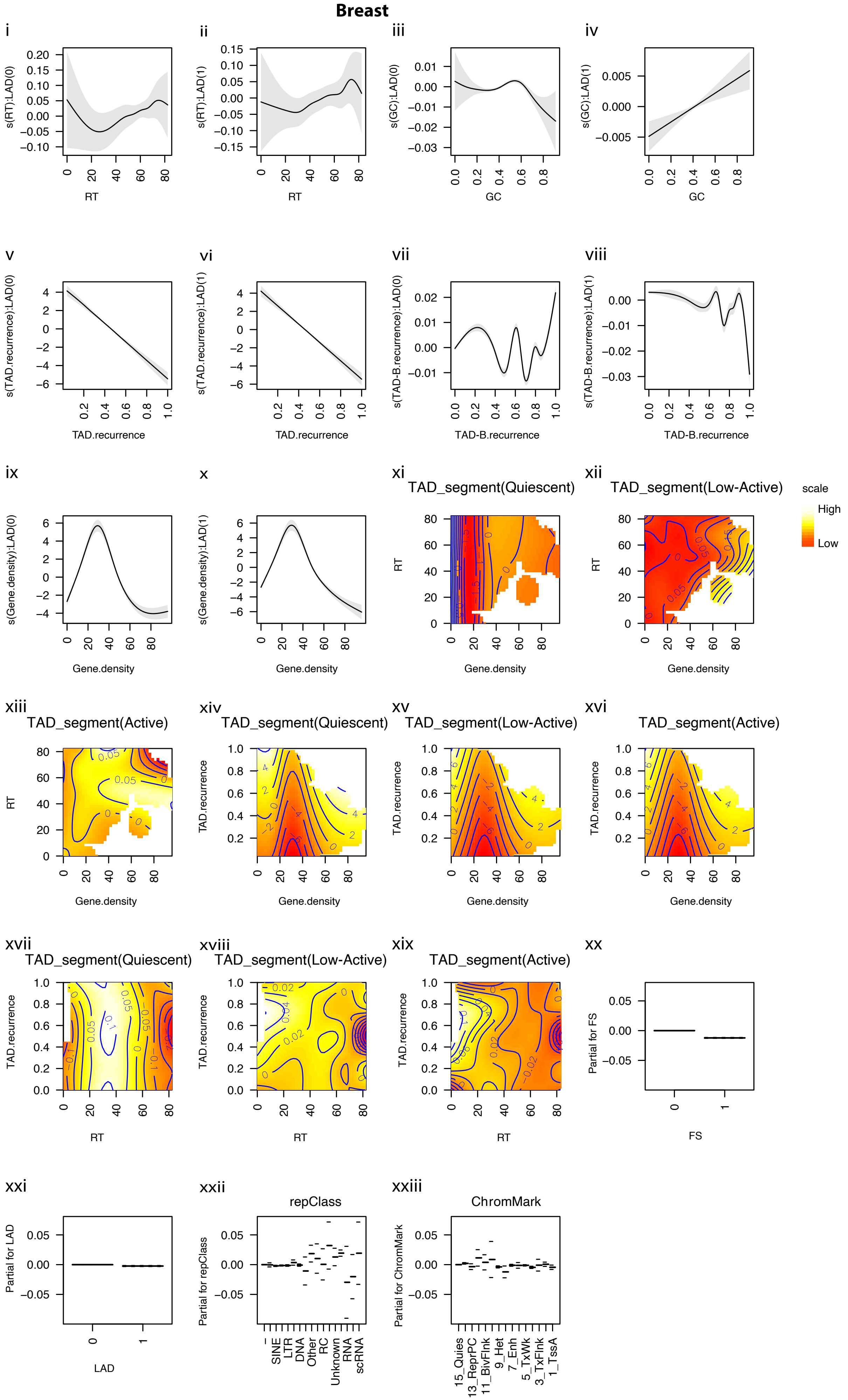

Supplementary Figure 3d

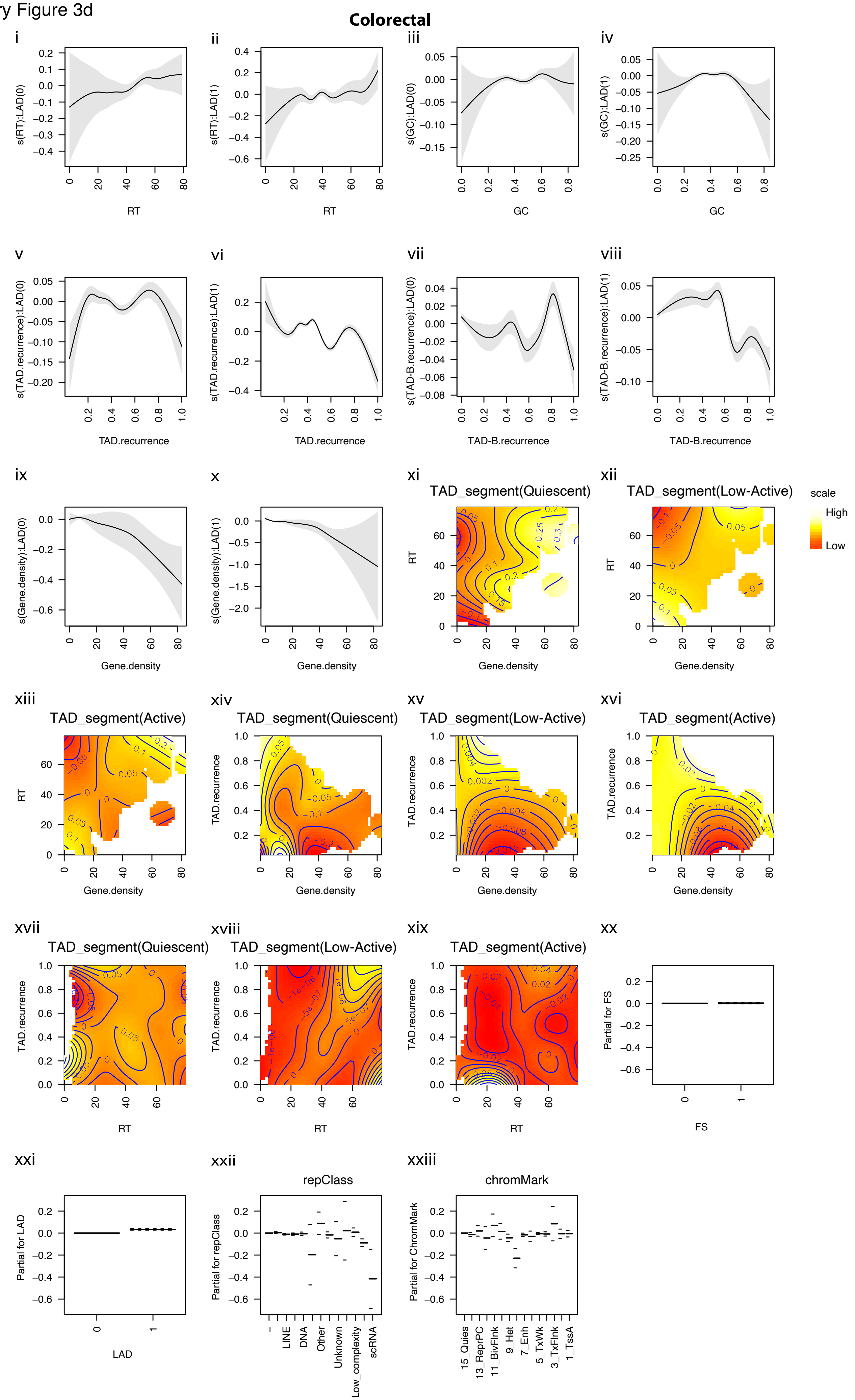

Supplementary Figure 3e

Esophagus

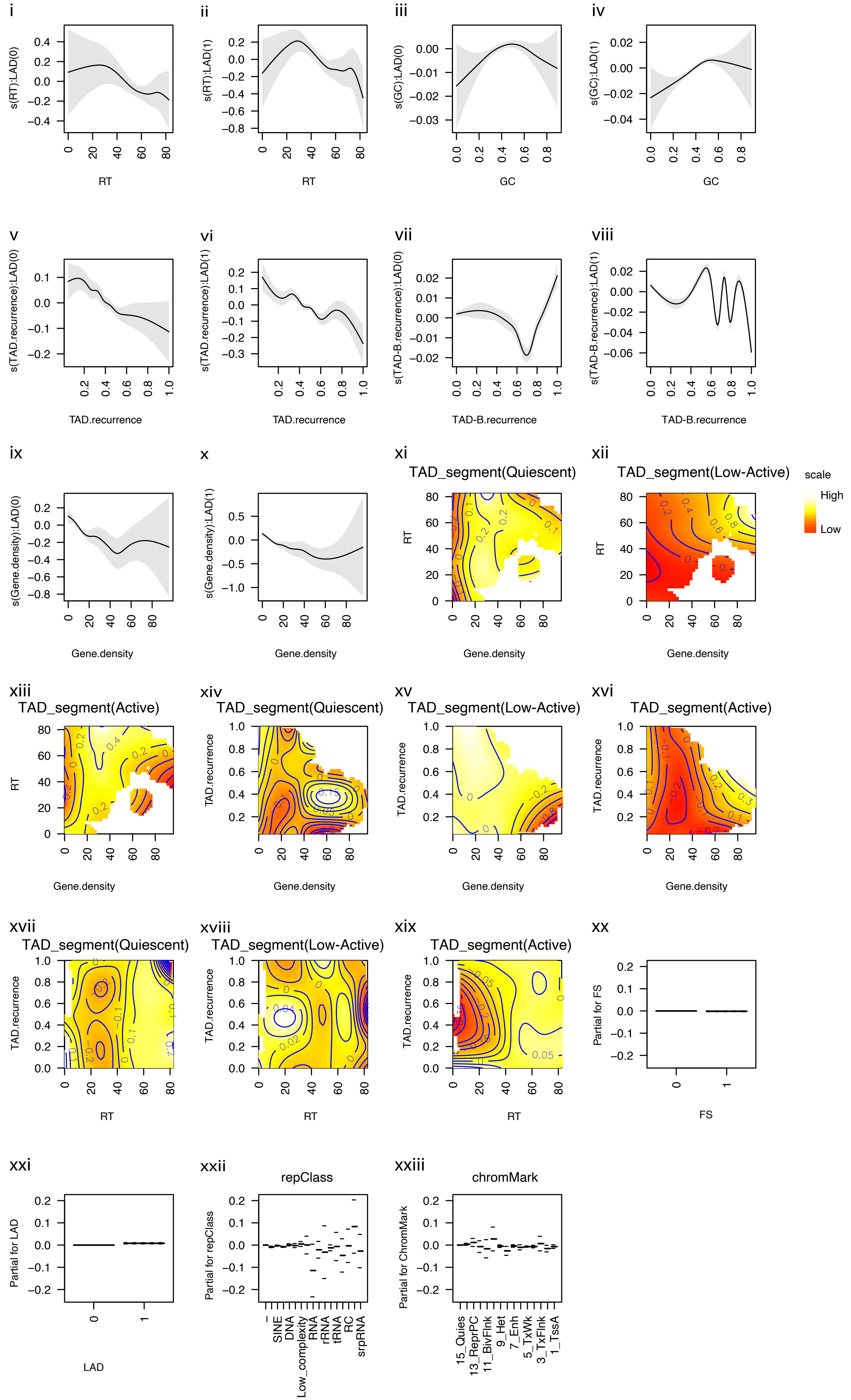

Supplementary Figure 3f

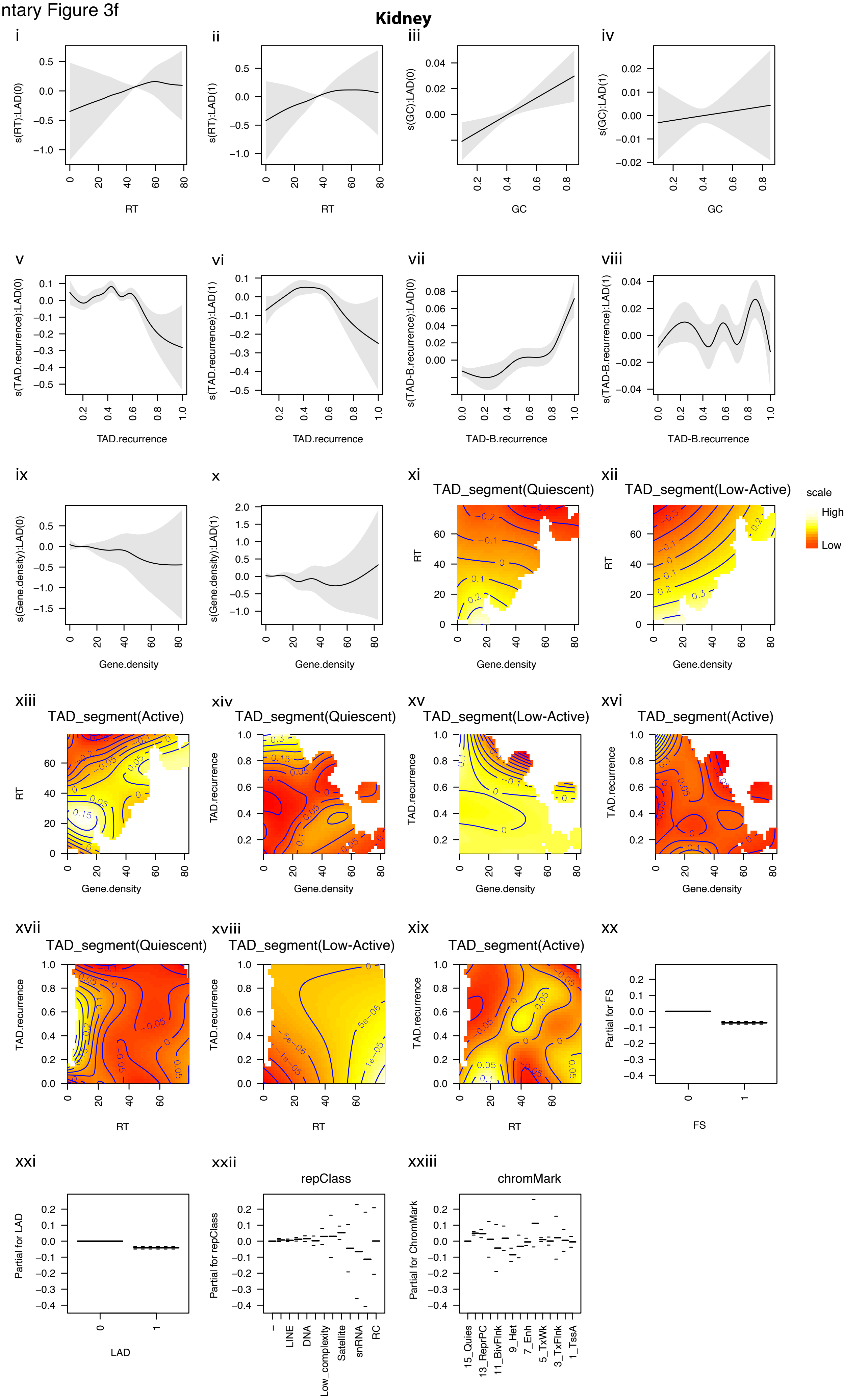

Supplementary Figure 3g

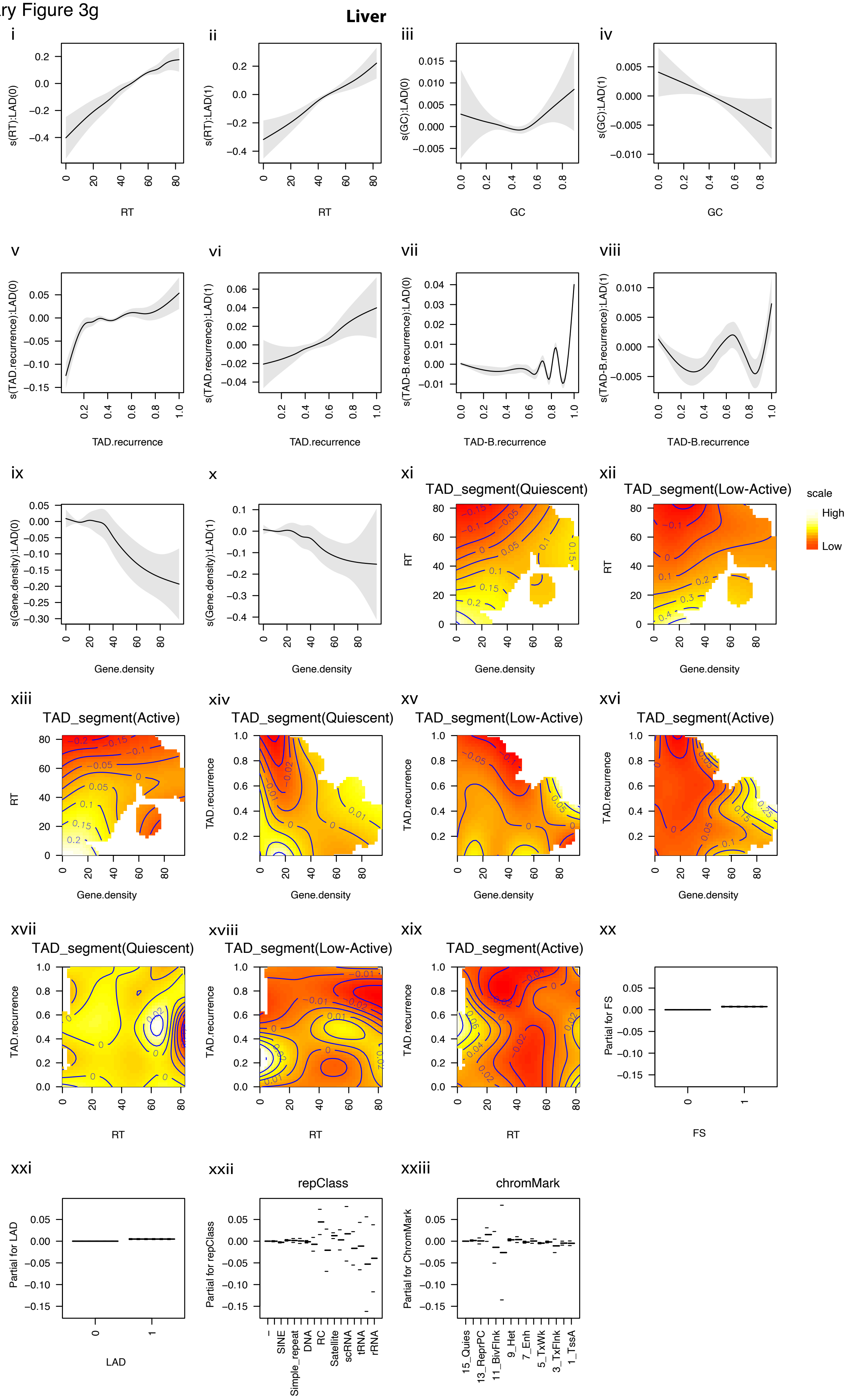

Supplementary Figure 3h

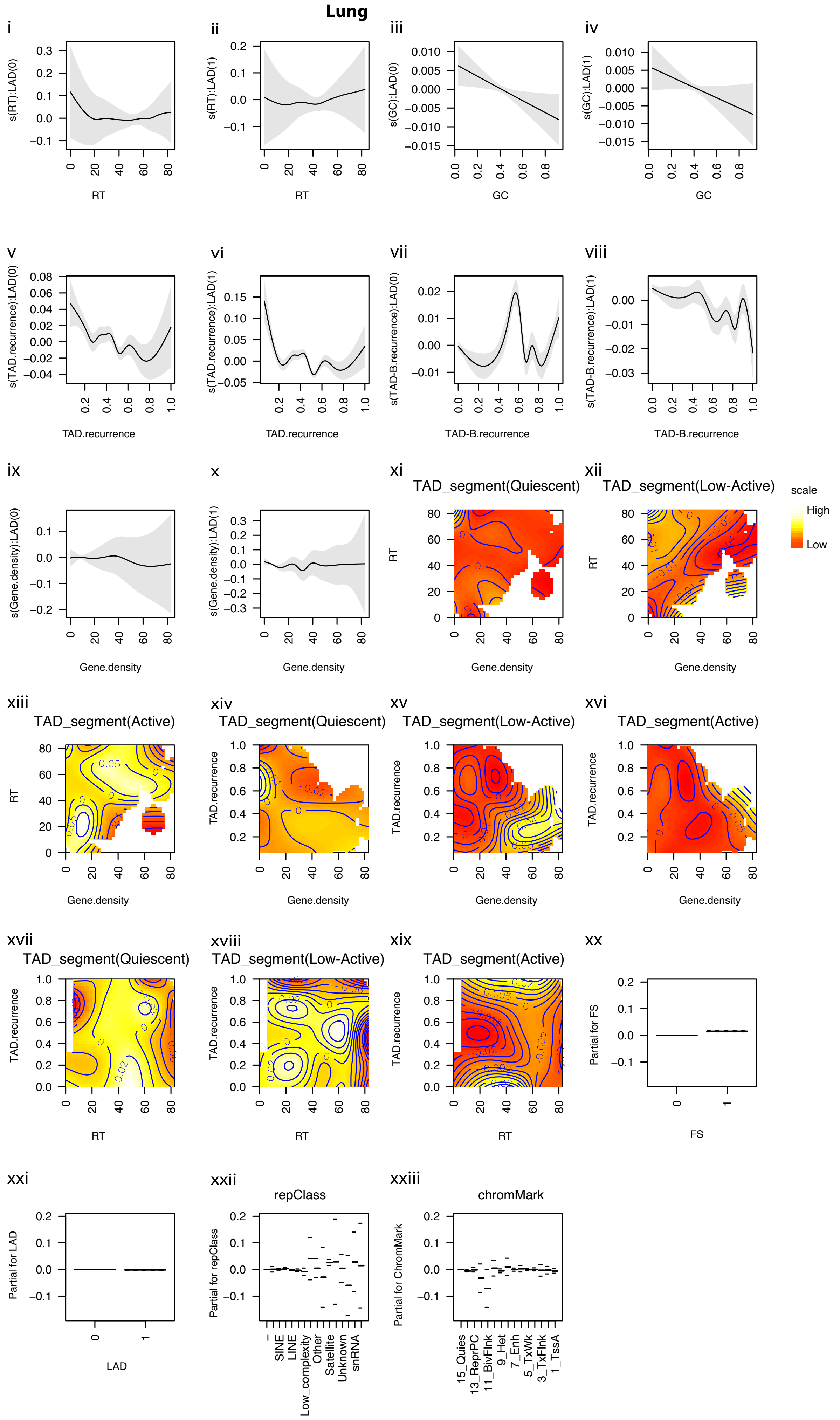

Supplementary Figure 3i

Lymph-nodes

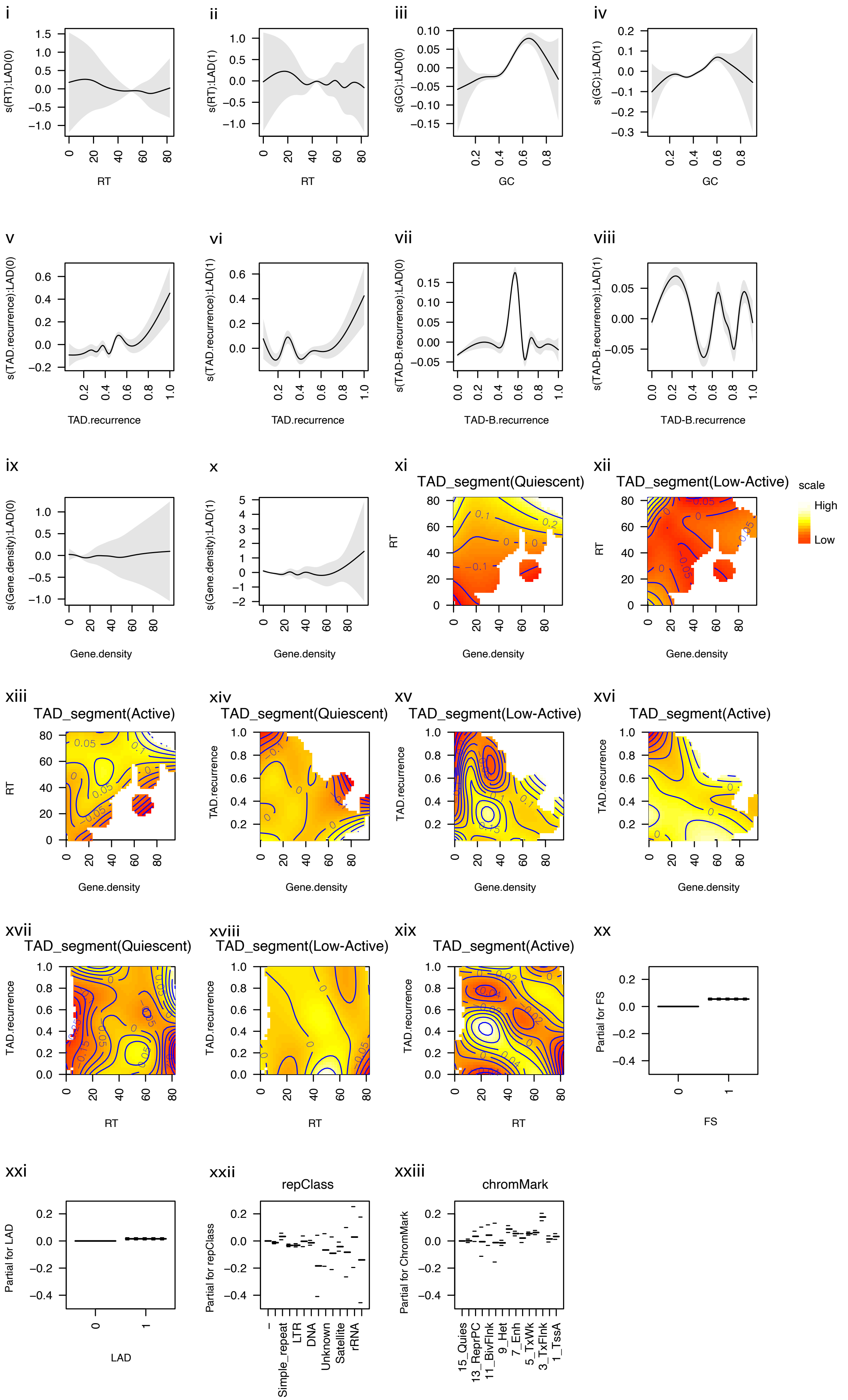

Supplementary Figure 3j

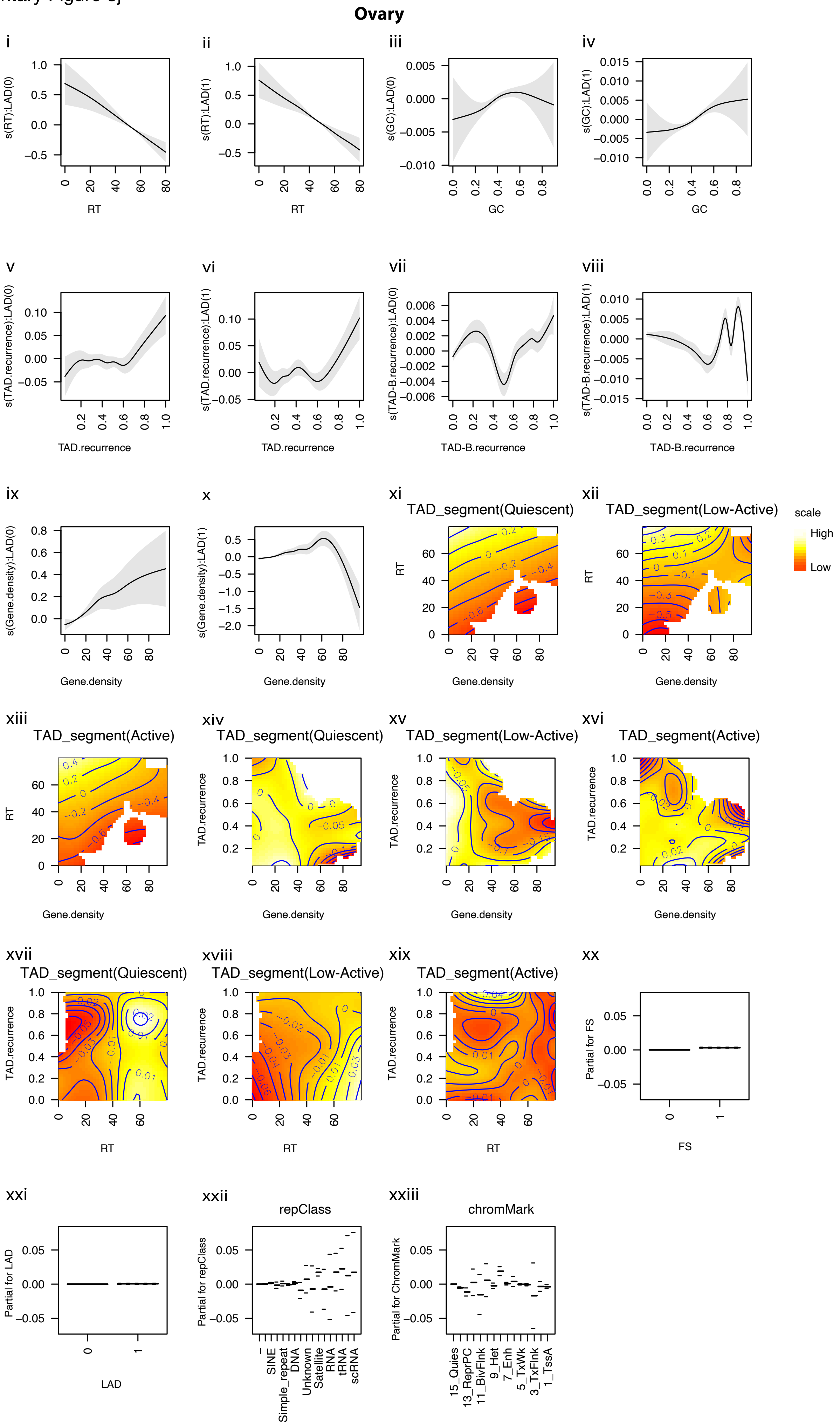

Supplementary Figure 3k

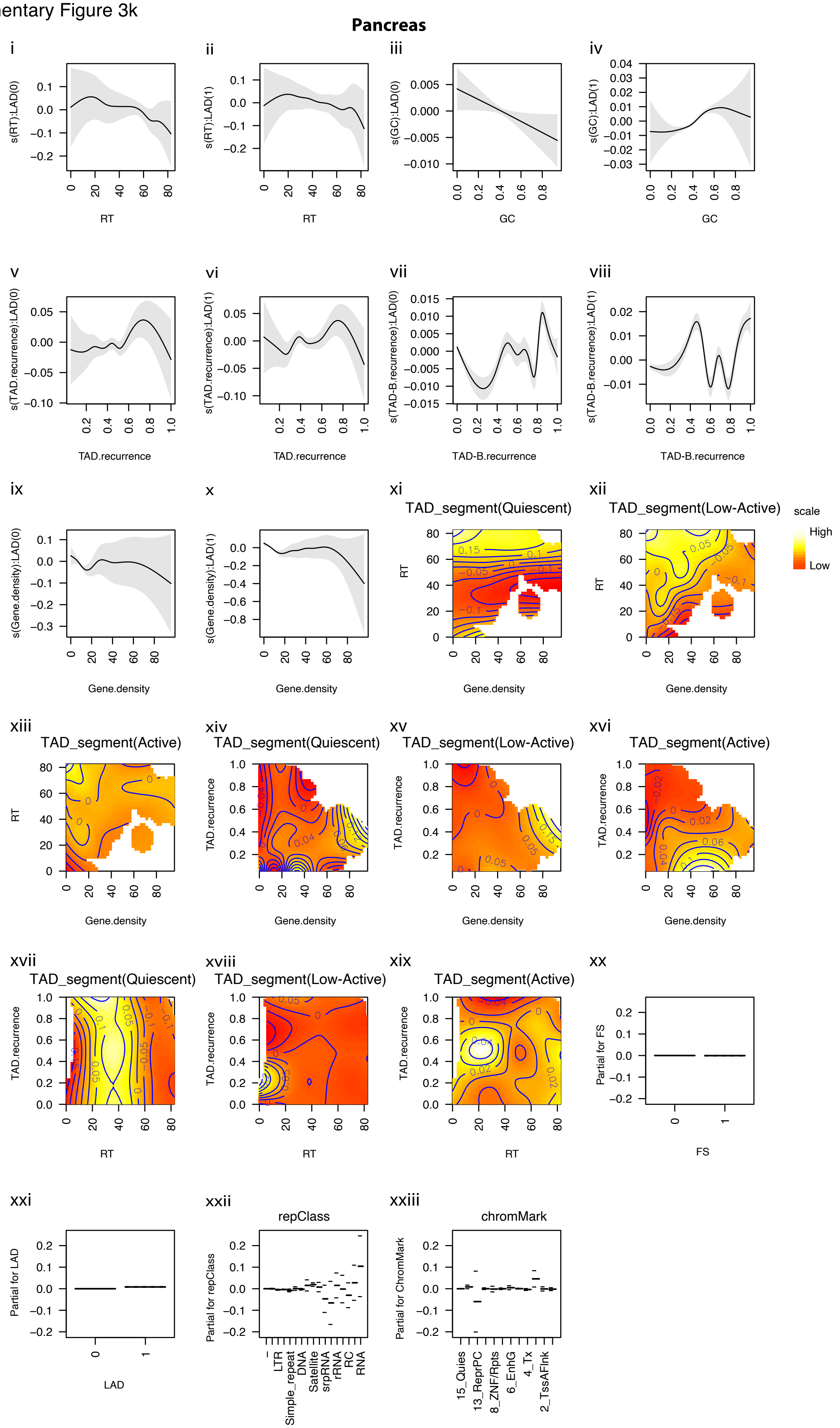

Supplementary Figure 3l

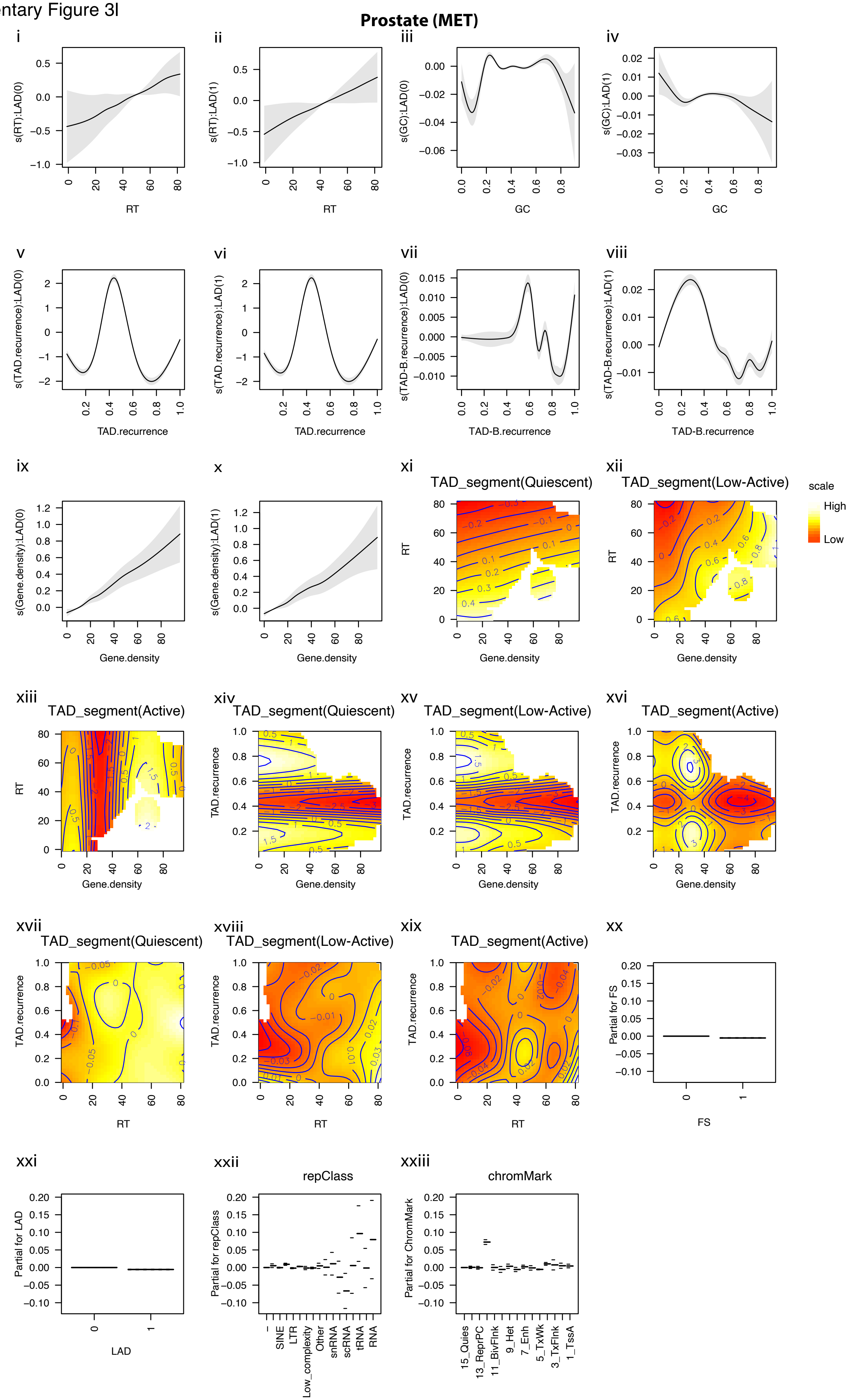

Supplementary Figure 3m

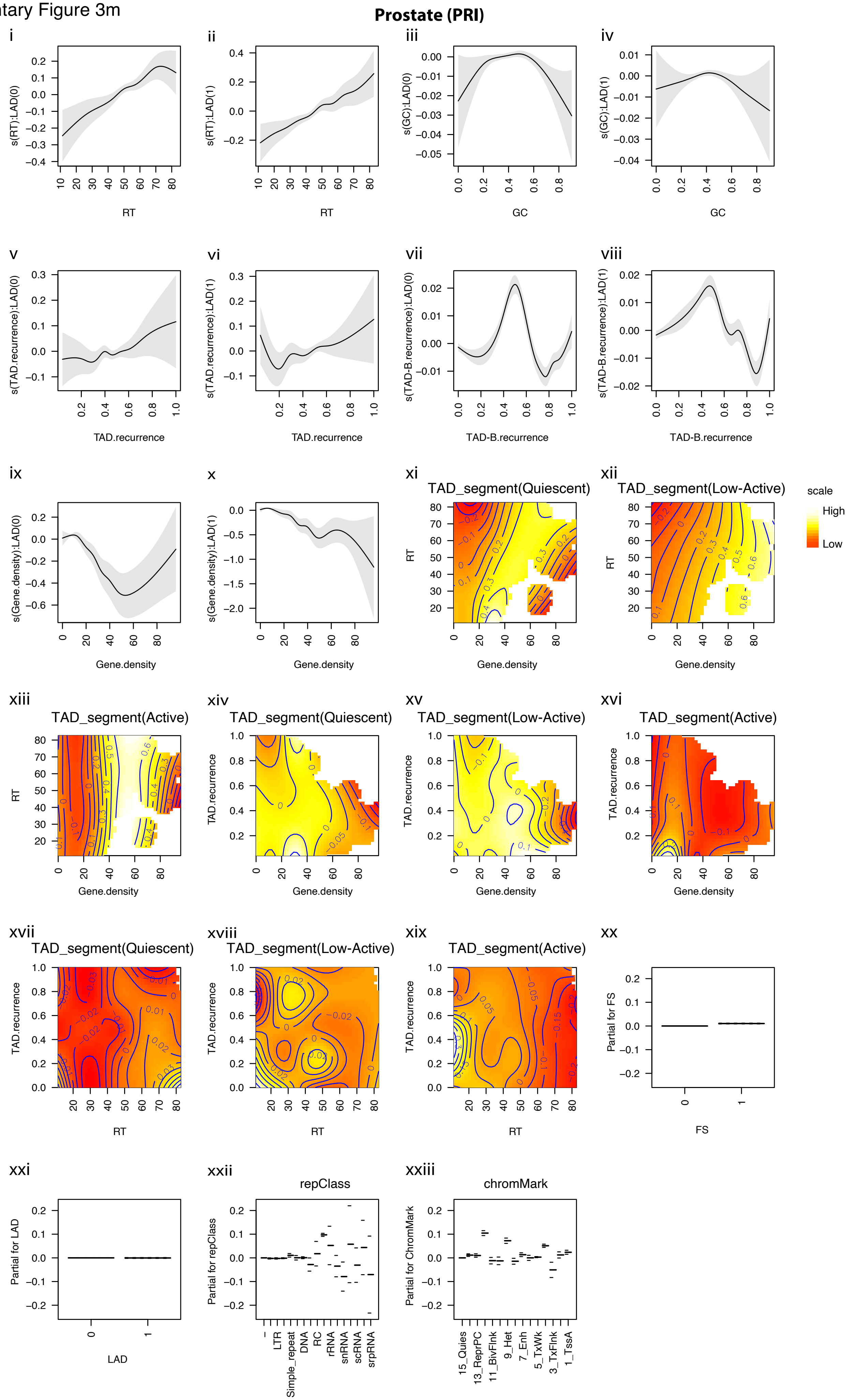

Supplementary Figure 3n

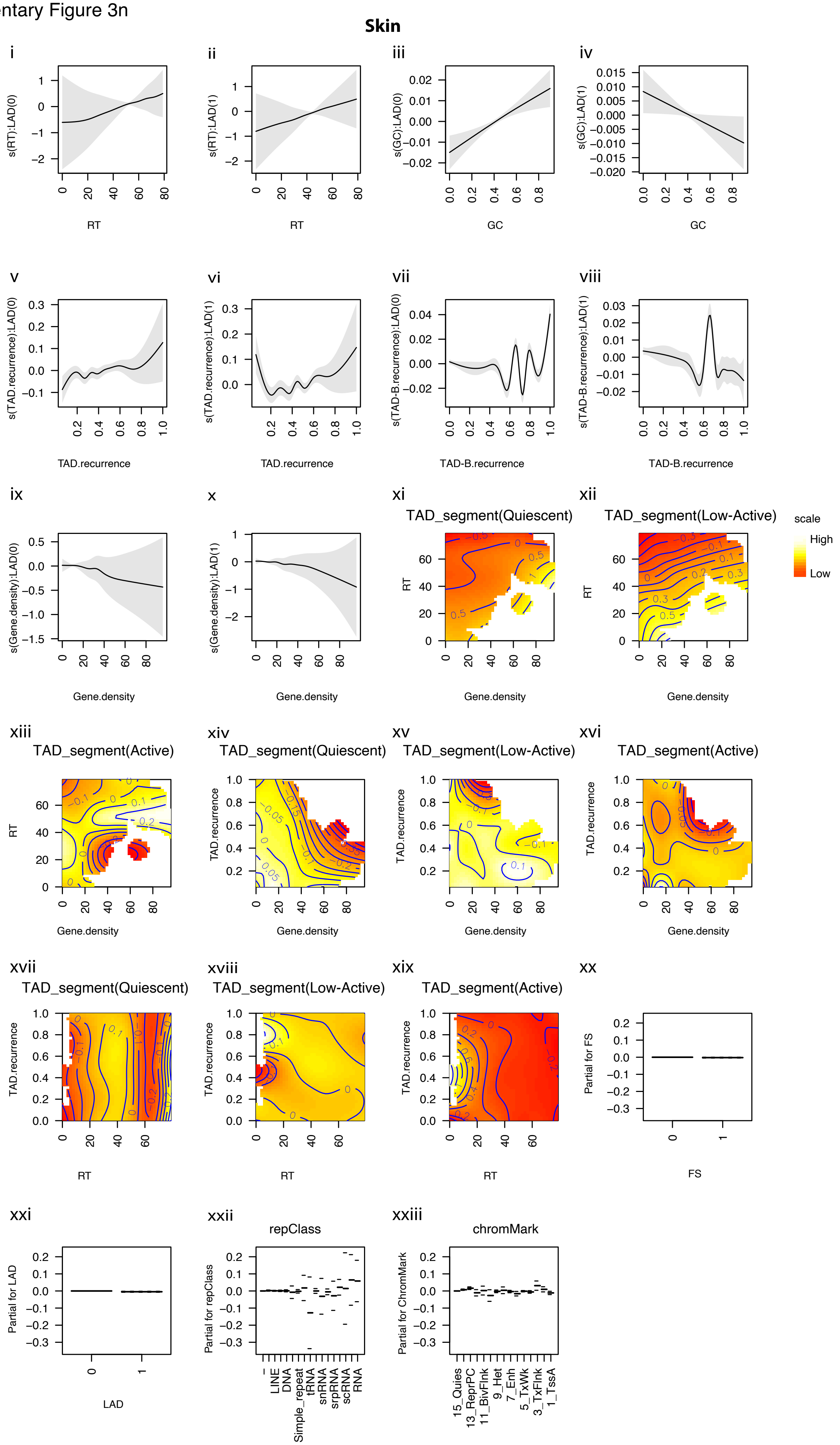

Supplementary Figure 3o

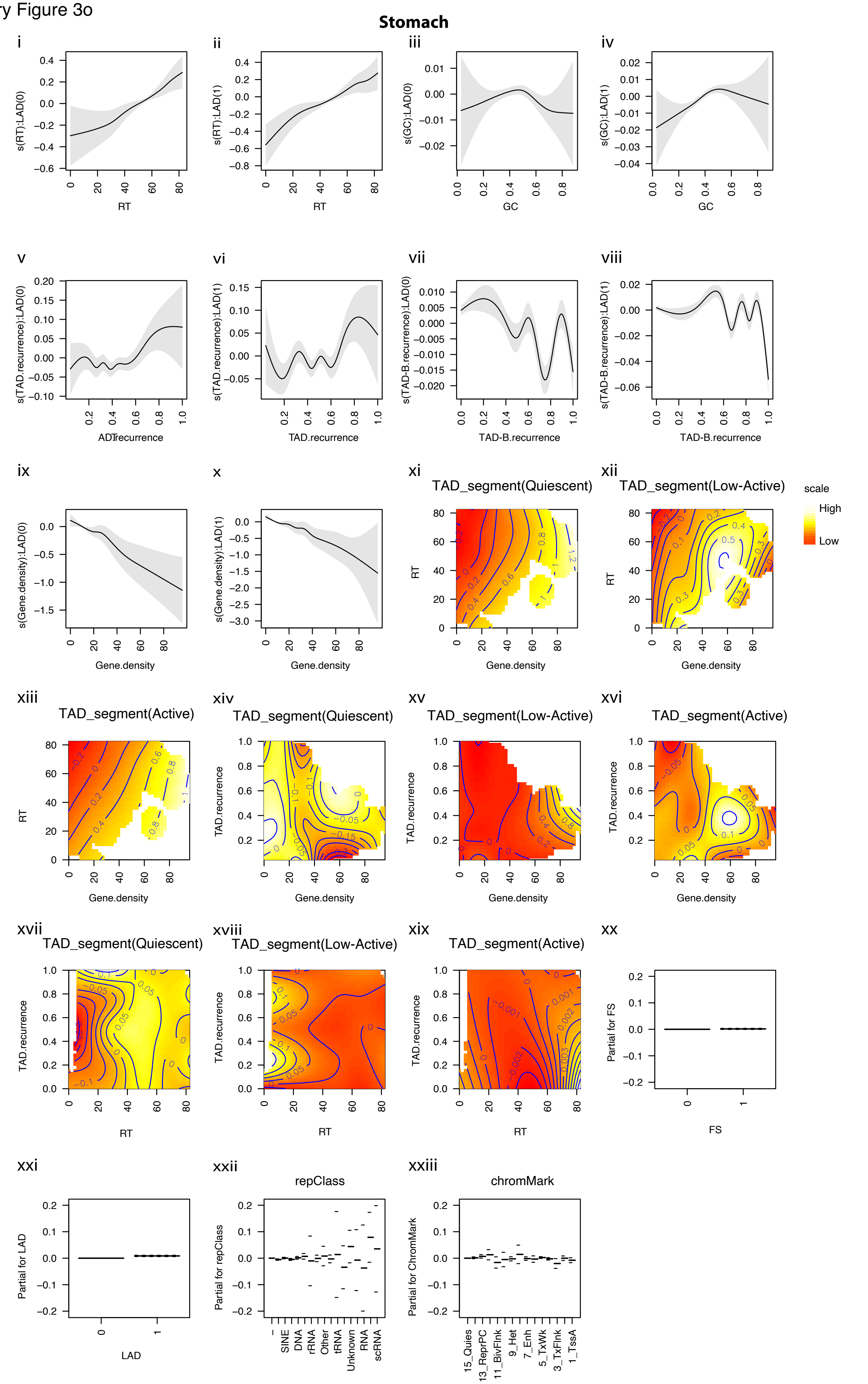

Supplementary Figure 3p

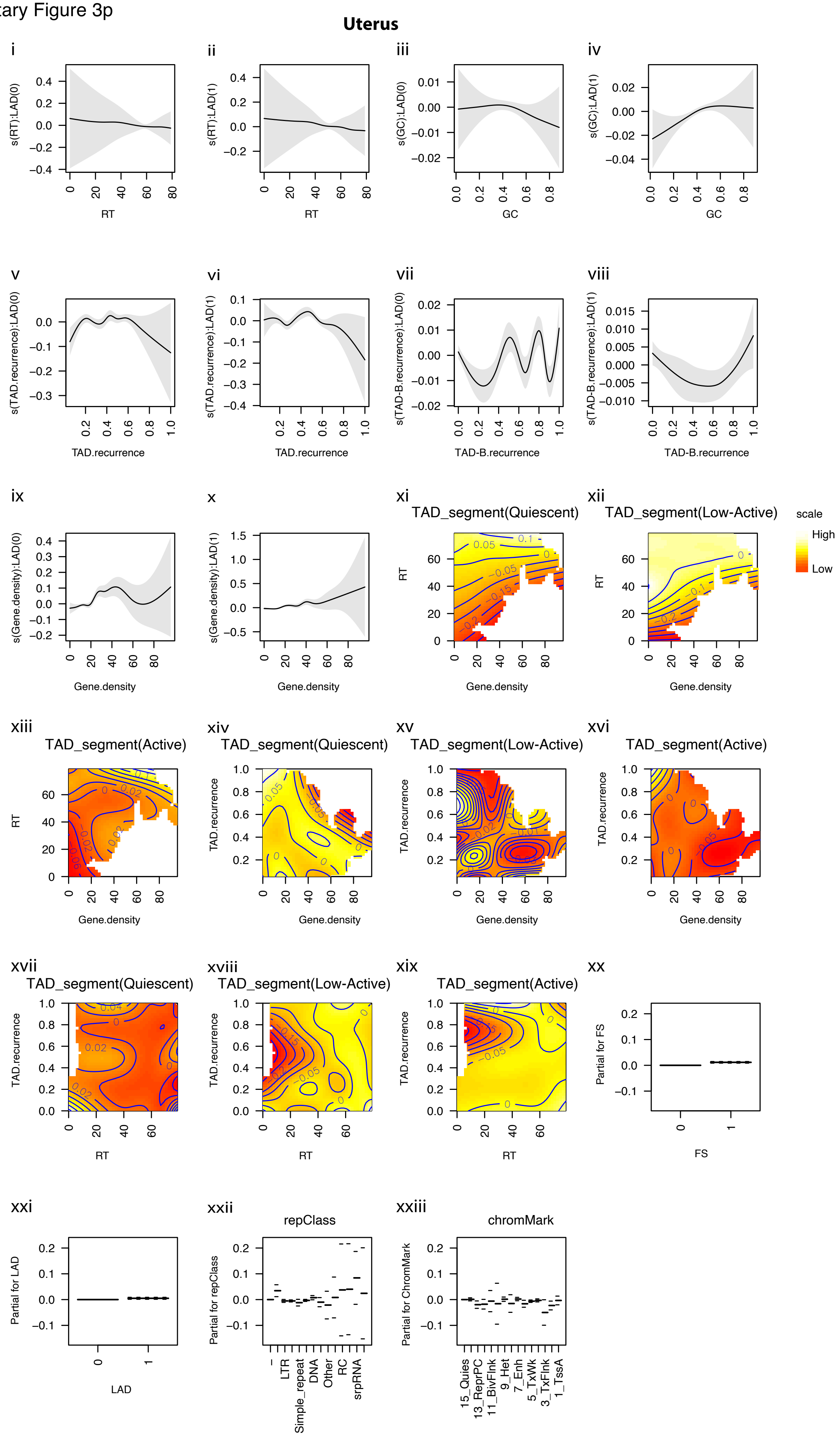

Supplementary Figure 3: Graphics of the GAM results.
The figure shows for each cohort panel (a-p), the graphics to analyze the partial contribution of each covariate to the GAM. The plots show the smooth function that represents the linear and non-linear partial contributions of RT (replication timing), GC, TAD.recurr and TAD-B.recurr for both within and outside LADs. The x-axis is the value of the covariate and the y-axis is the corresponding partial effect from its contribution function. The grey shade area displays two standard errors above and below the GAM estimate of the smooth curve. The plots also show the 2D graphics of the partial contribution of the interplay between (gene.density vs. TAD.recurr), (gene.density vs. RT) and (RT vs. TAD.recurr). Plots are shown for all the TAD segment classes (active, low-active, quiescent/Het). The scale from light yellow to red represents partial contribution for higher to lower values of the distribution. The plots for the contribution of covariates with binary values (presence or absence) are also shown (fragile sites, presence in LAD, repeat class, chromatin marks). The two standard errors above and below are represented by dashed line (upper and lower).

Supplementary Figure 4

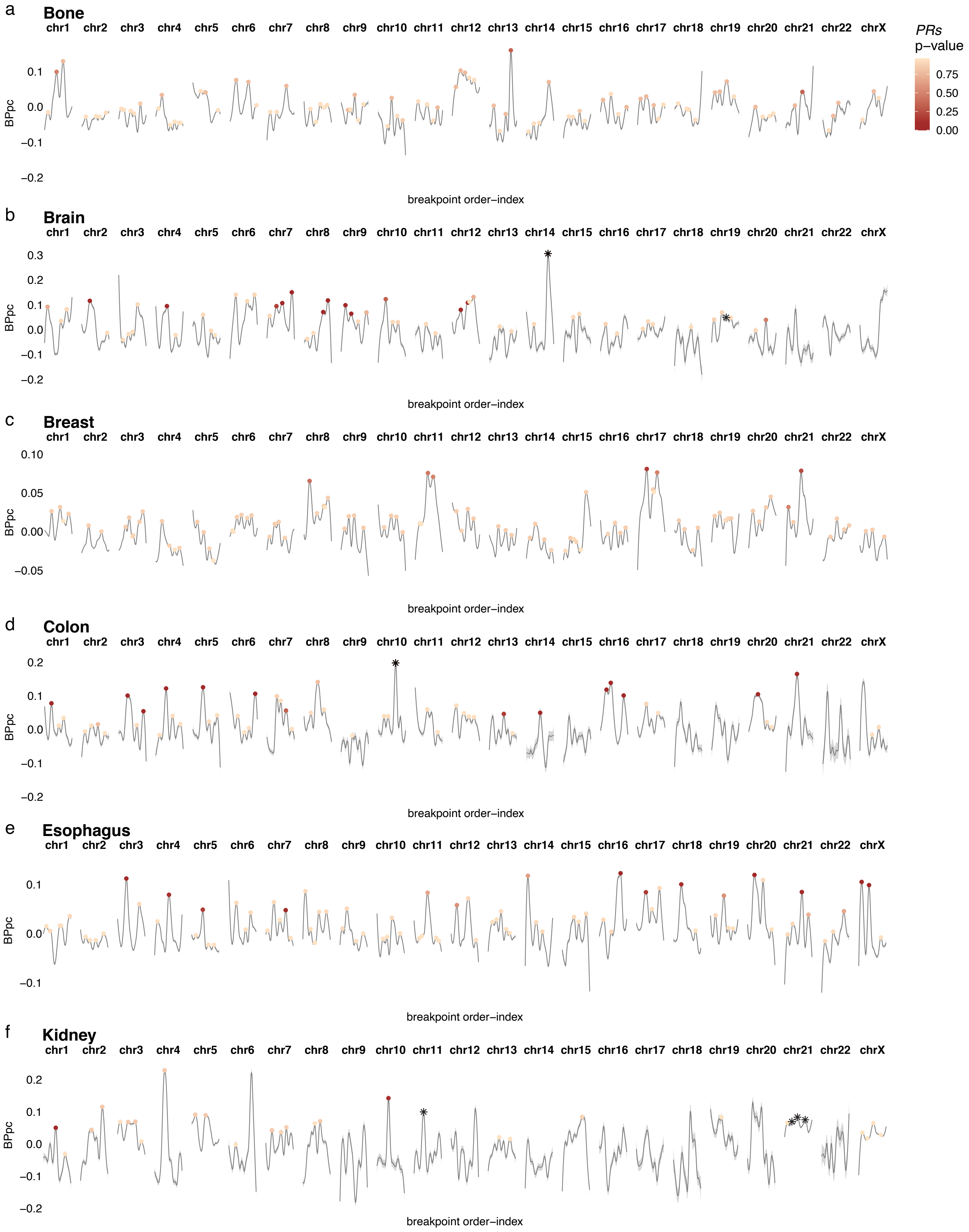

Supplementary Figure 4 (Cont.)

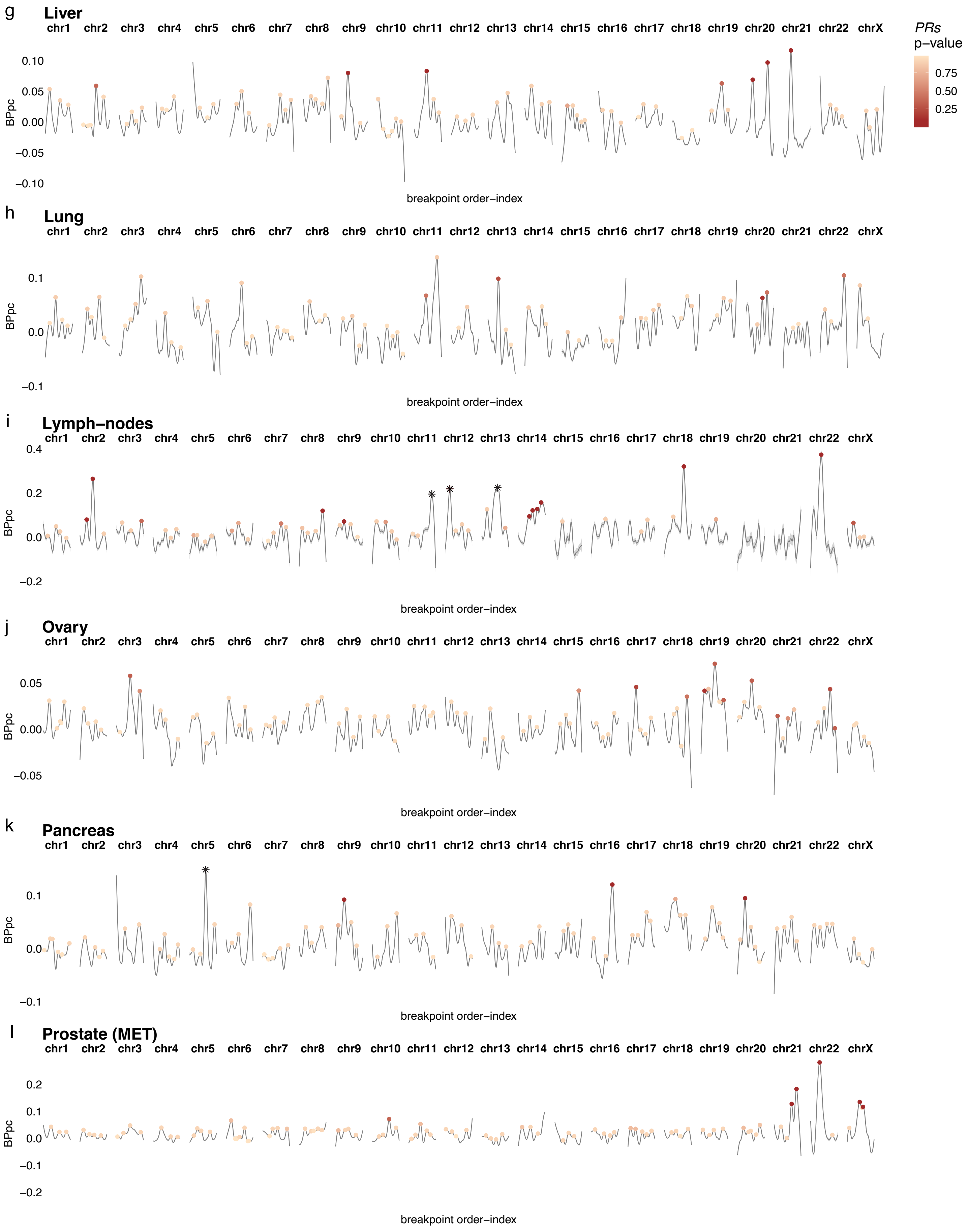

Supplementary Figure 4 (Cont.)

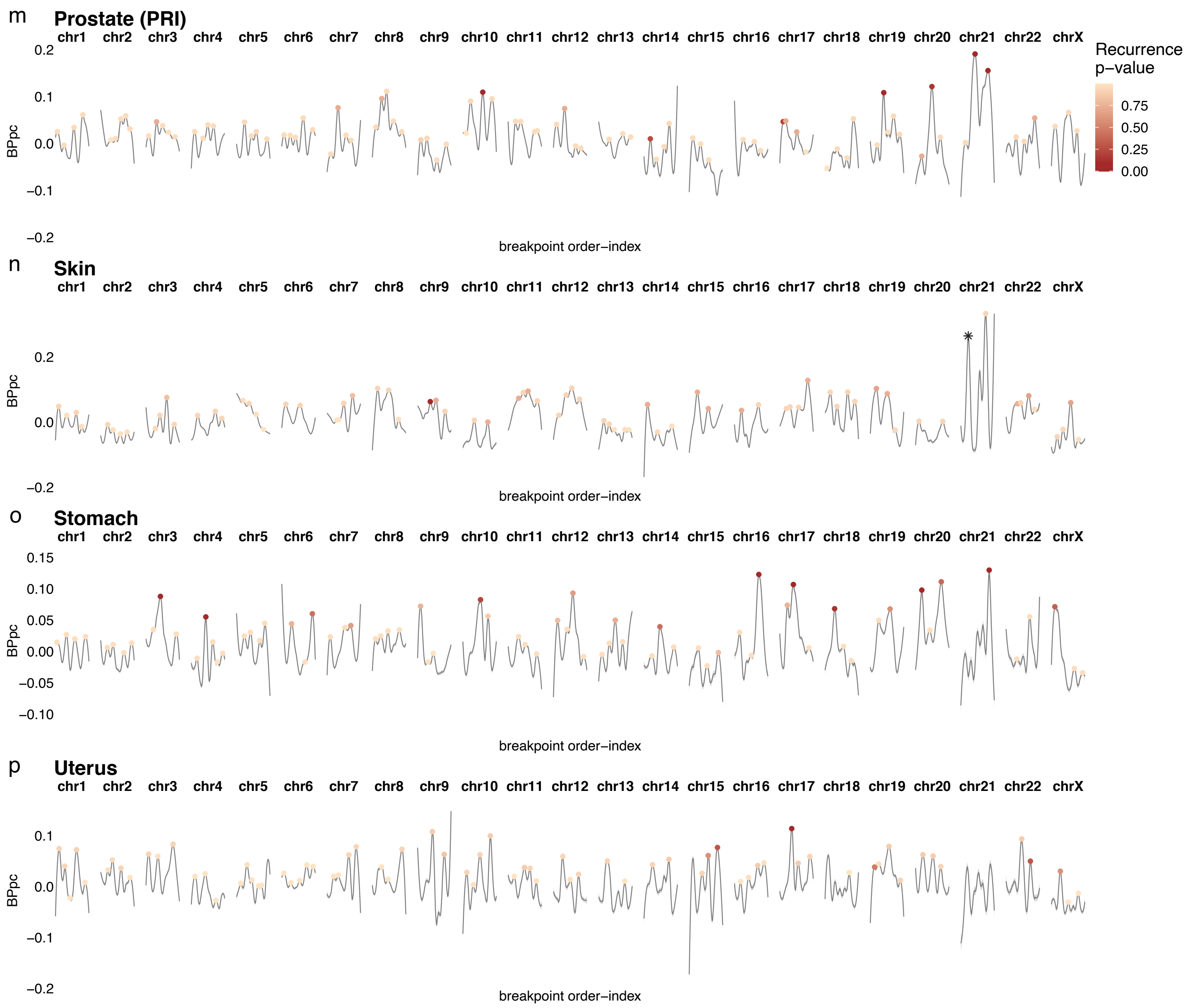

Supplementary Figure 4: Graphic of the cancer-specific BPpc for each cohort panel (a-p).
Each peak shows a dot colored using empirical p-value of significance for the test of fit for the Gamma distribution of corresponding peak recurrence score (PRs). The scale from light to dark brown represents higher to lower p-value. The grey shade around the curves (loess smoothing) displays a 95% confidence interval.

Supplementary Figure 5

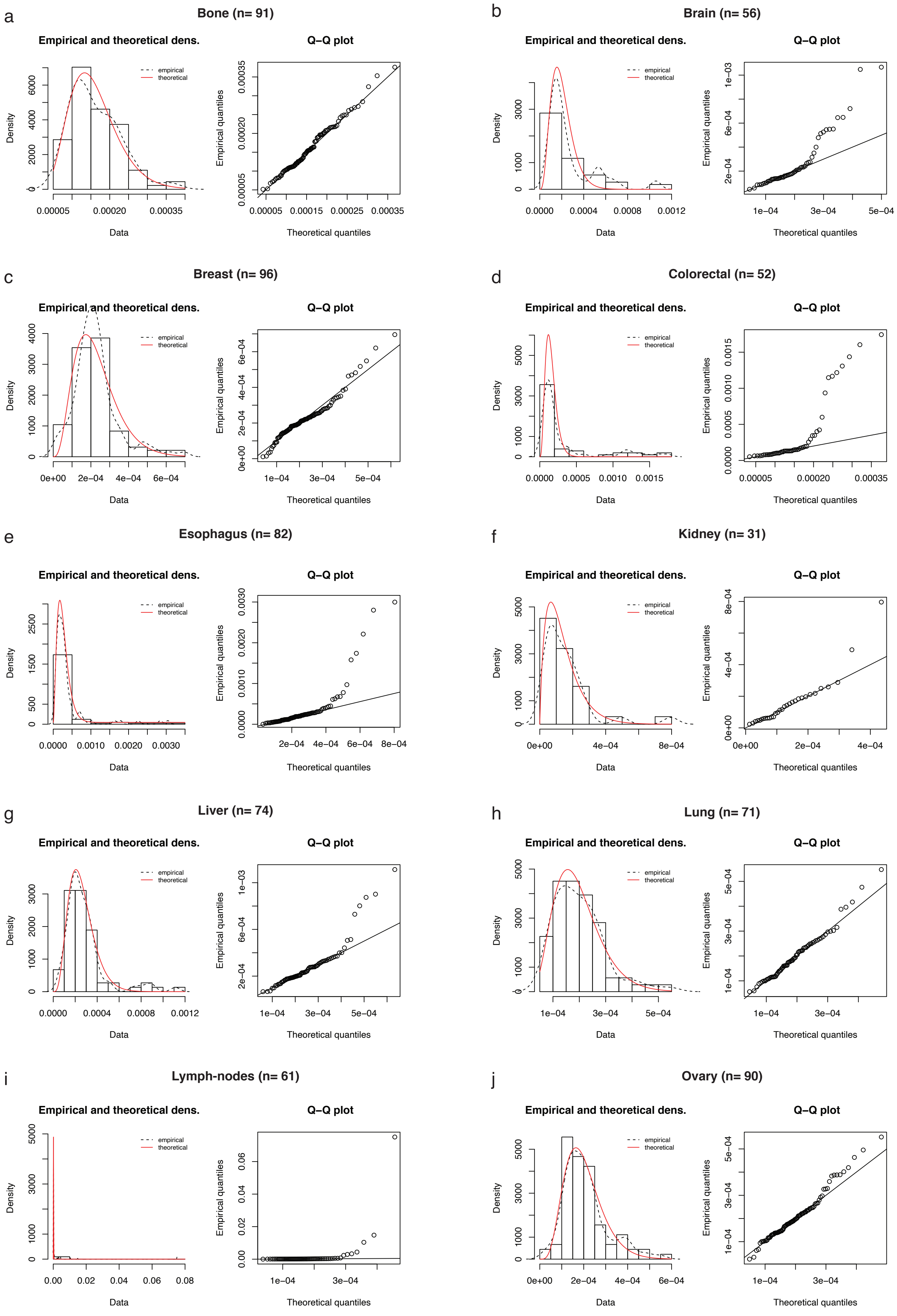

Supplementary Figure 5 (Cont.)

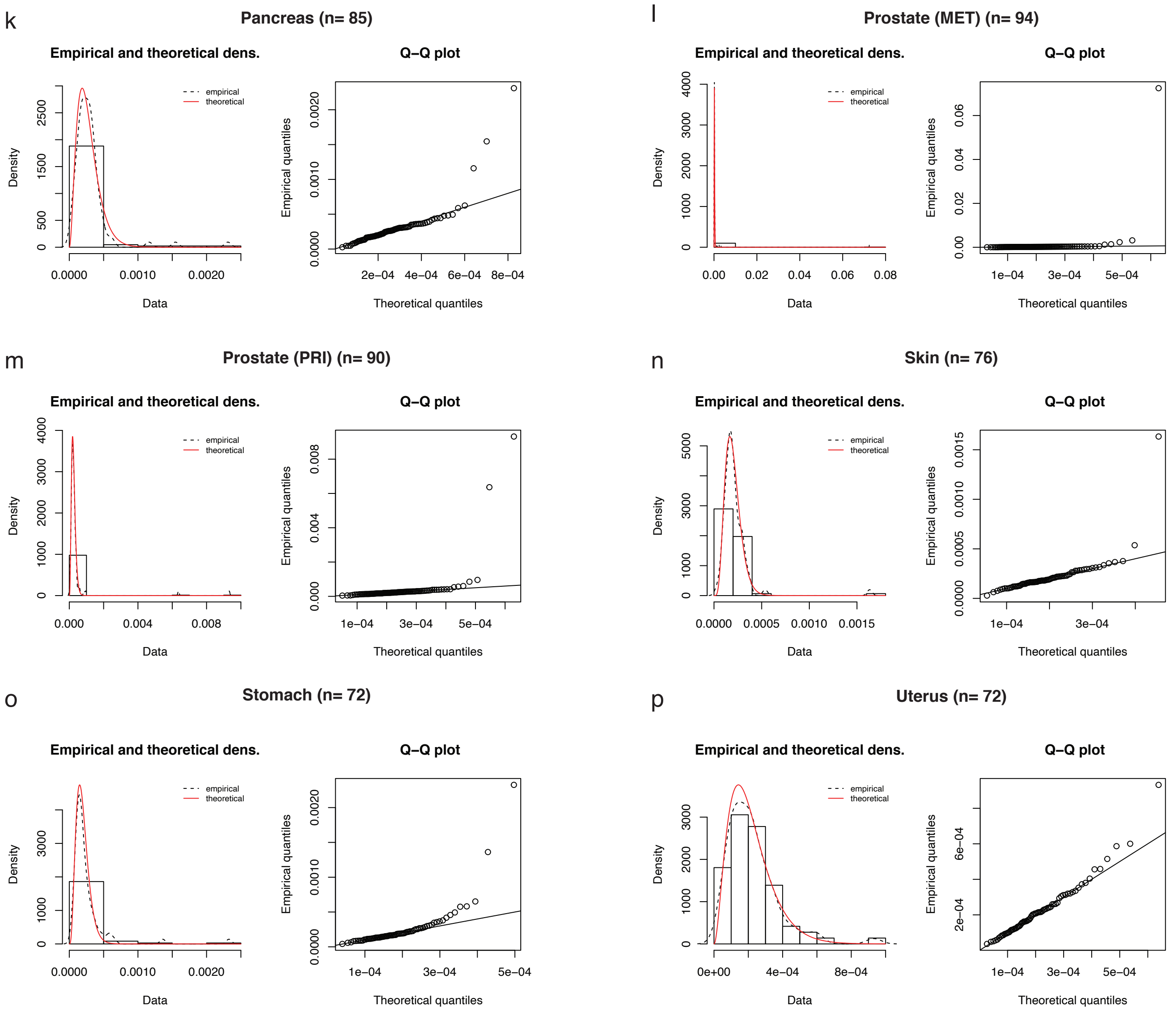

Supplementary Figure 5: Distribution of the peak recurrence scores (PRs) and their probabilities. For each cohort panel (a-p), the graphics show the empirical and theoretical density of PRs and the QQ-plots for the test of fit for the Gamma distribution to assess the peaks of significantly recurrent rearrangements. The total number of independent peaks (n) is annotated for each cohort.

Supplementary Figure 6

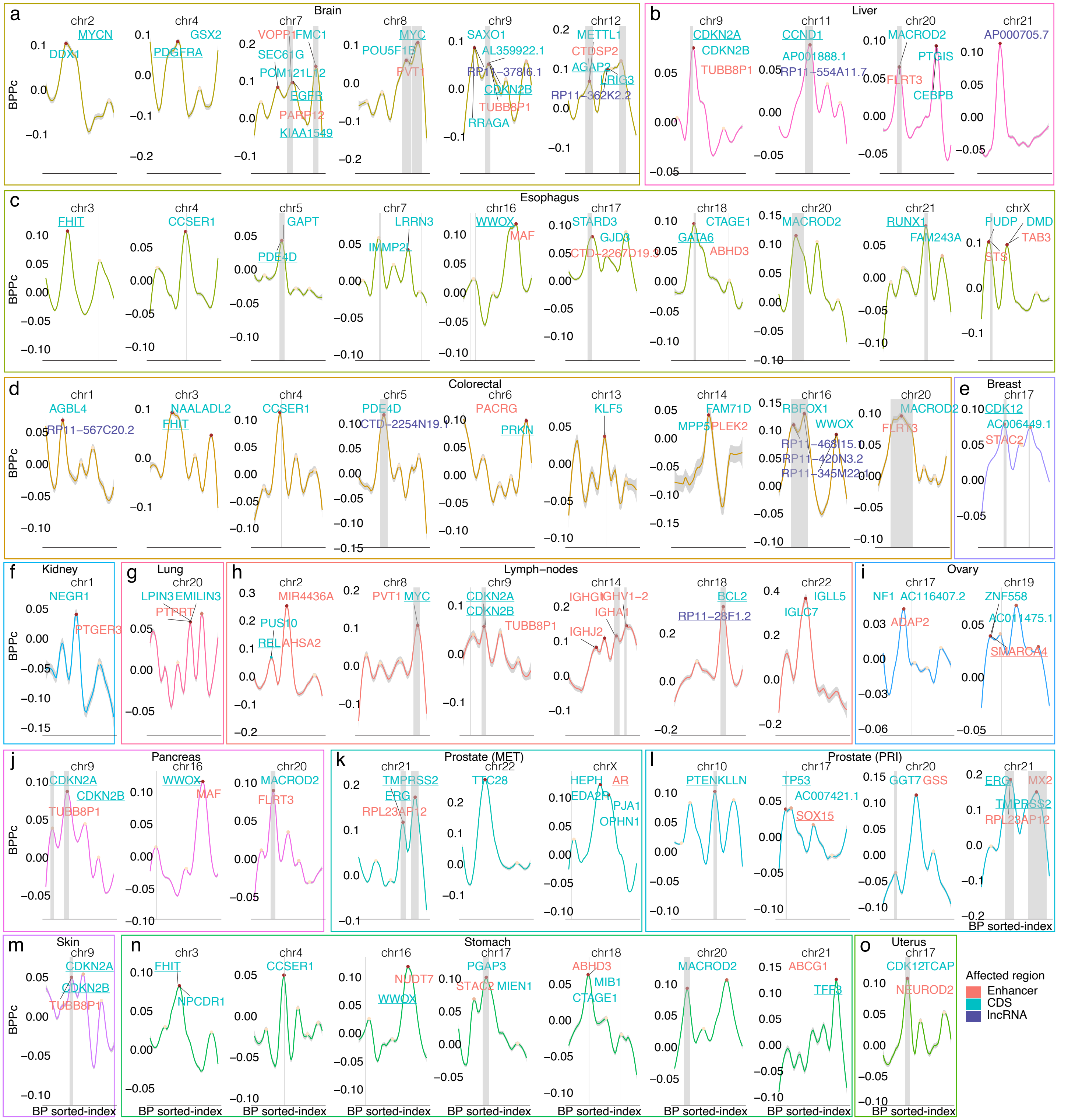

Significant peaks and the predicted driver candidates.
The figure shows for each cohort panel (a-o), the BPpc for each chromosome with significant peaks annotated with the predicted driver candidates in the region. Drivers in green are affected in the coding sequence and drivers in red are affected in their enhancer regulatory regions. The grey rectangles show the regions that overlap with significantly high-density loci detected by the approach of PCAWG using a non-cancer-specific method.

Supplementary Figure 7

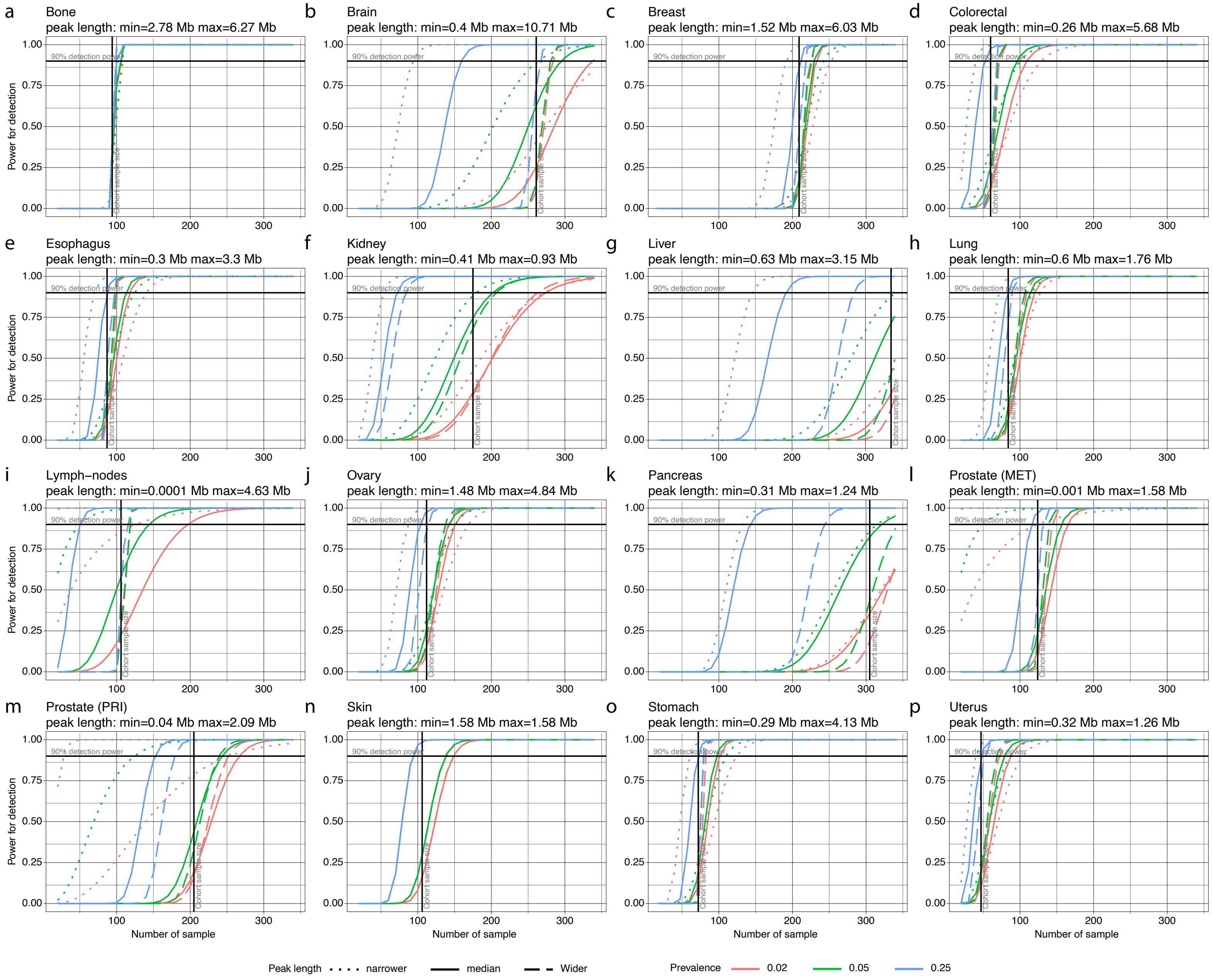

Detection power of the significant peaks.
For each cohort panel (a-p), the scale of prevalence shows peaks of 2% frequency in red, 5% in green and 25% in blue.
The genomic range of the peaks is annotated with dotted-line for the narrow peaks, solid line for the median length peaks and dashed-line for wide peaks for each cohort. The horizontal black line is the threshold of 90% detection power. The vertical black line is the cohort sample size.

Supplementary Figure 8

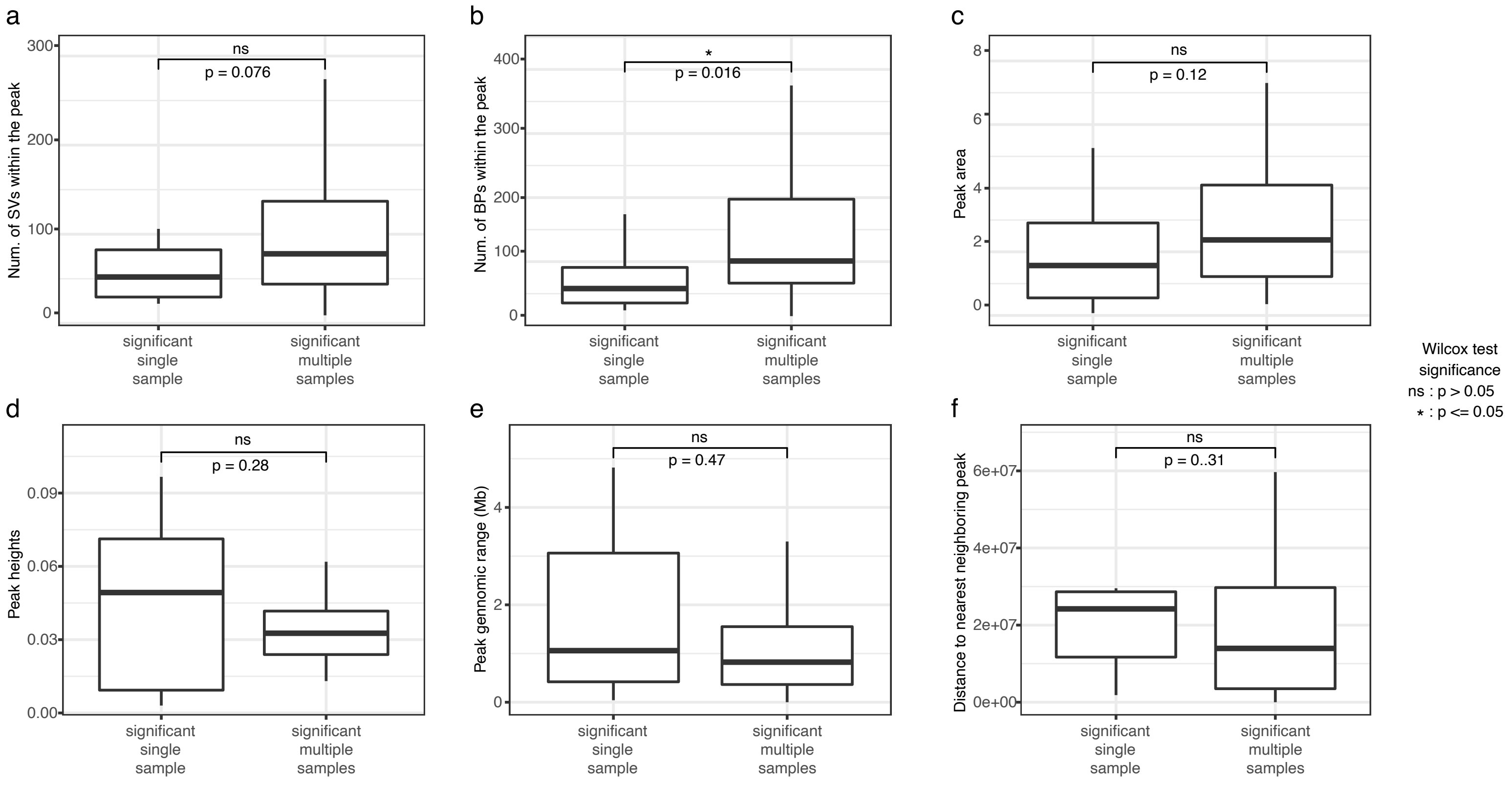

BPpc features comparison between the significant single-sample peaks and the significant multiple-samples peaks applying two-sided Wilcoxon rank sum test (a) Number of SVs for each peak within the subgroup. (b) Number of breakpoints for each peak within the subgroup. (c) Area of the peaks within each subgroup. (d) Peak height for each subgroup. (e) Length of the genomic range for each peak within the subgroup. (f) Distance of the peaks to the closest neighboring peaks per subgroup. The middle hinge corresponds to the median. The lower and upper hinges correspond to the first and third quartiles (the 25th and 75th percentiles). The upper whisker extends from the hinge to the largest value no further than 1.5 * IQR from the hinge, and the lower whisker extends from the hinge to the smallest value at most 1.5 * IQR of the hinge (IQR is the inter-quartile range, or distance between the first and third quartiles). Data beyond the end of the whiskers are plotted individually as outlier points.

Supplementary Figure 9

a Scheme of the TAD segments of different recurrence

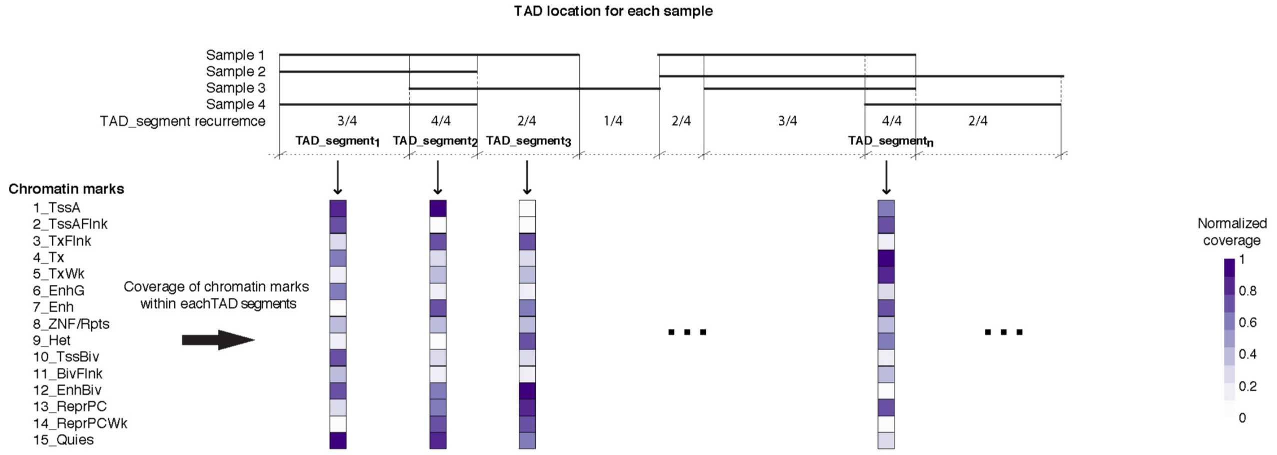

b Result of the chromatin marks coverage within TAD_segment for each cohort

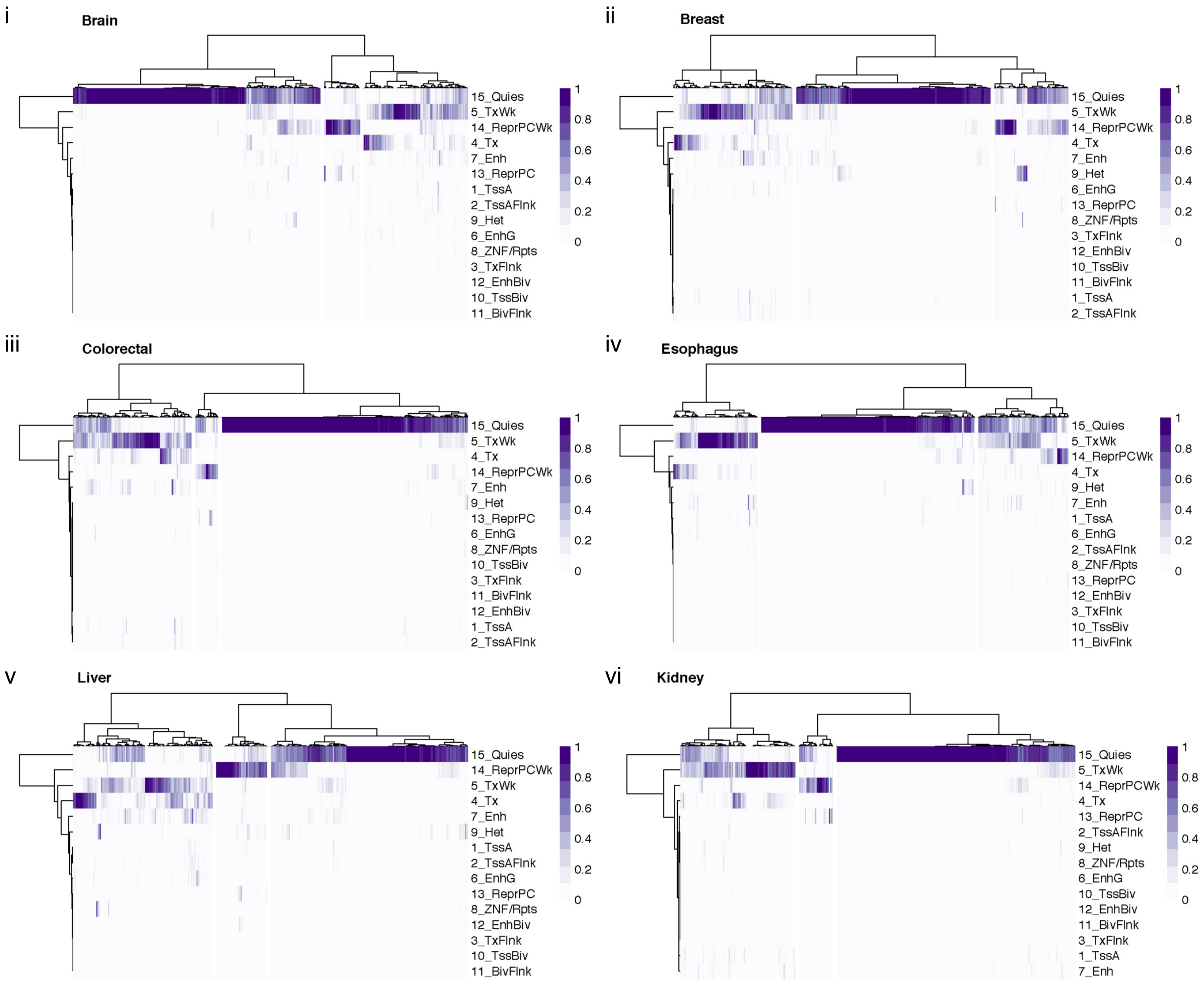

Supplementary Figure 9b (Cont.)

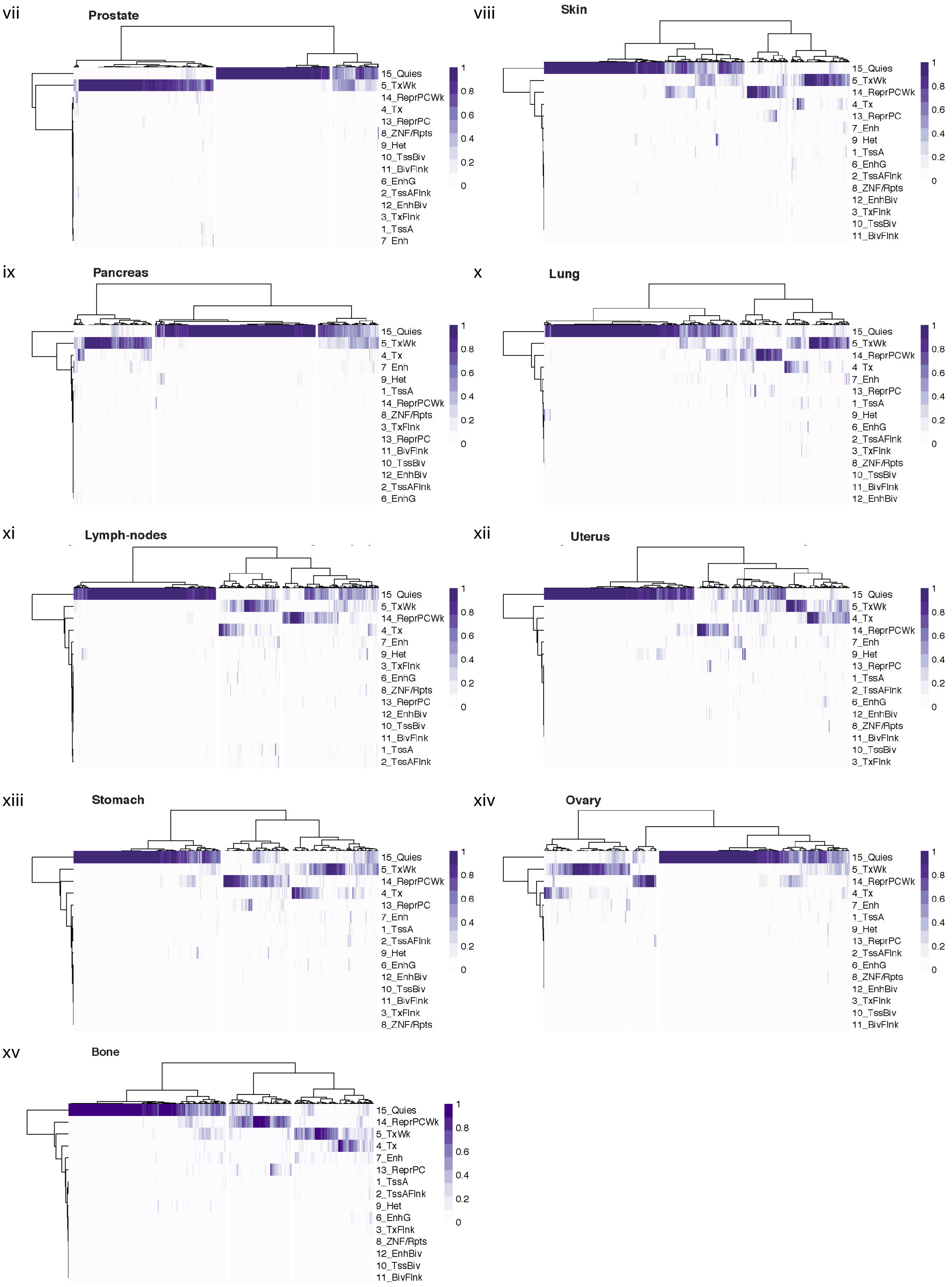

Supplementary Figure 9: Description of TAD segments and their classification based on coverage of chromatin marks. (a) Schematic representation of the process computing the chromatin marks' coverage for each TAD segments with different recurrence in samples (b) Plots for each cancer cohort show the heatmap of the enrichment of chromatin marks for each TAD segment. The columns correspond to the different chromatin marks and the rows correspond to TAD segment. The columns are clustered and split into three blocks. The scale goes from light to dark purple in ascending enrichment.

Supplementary Figure 10

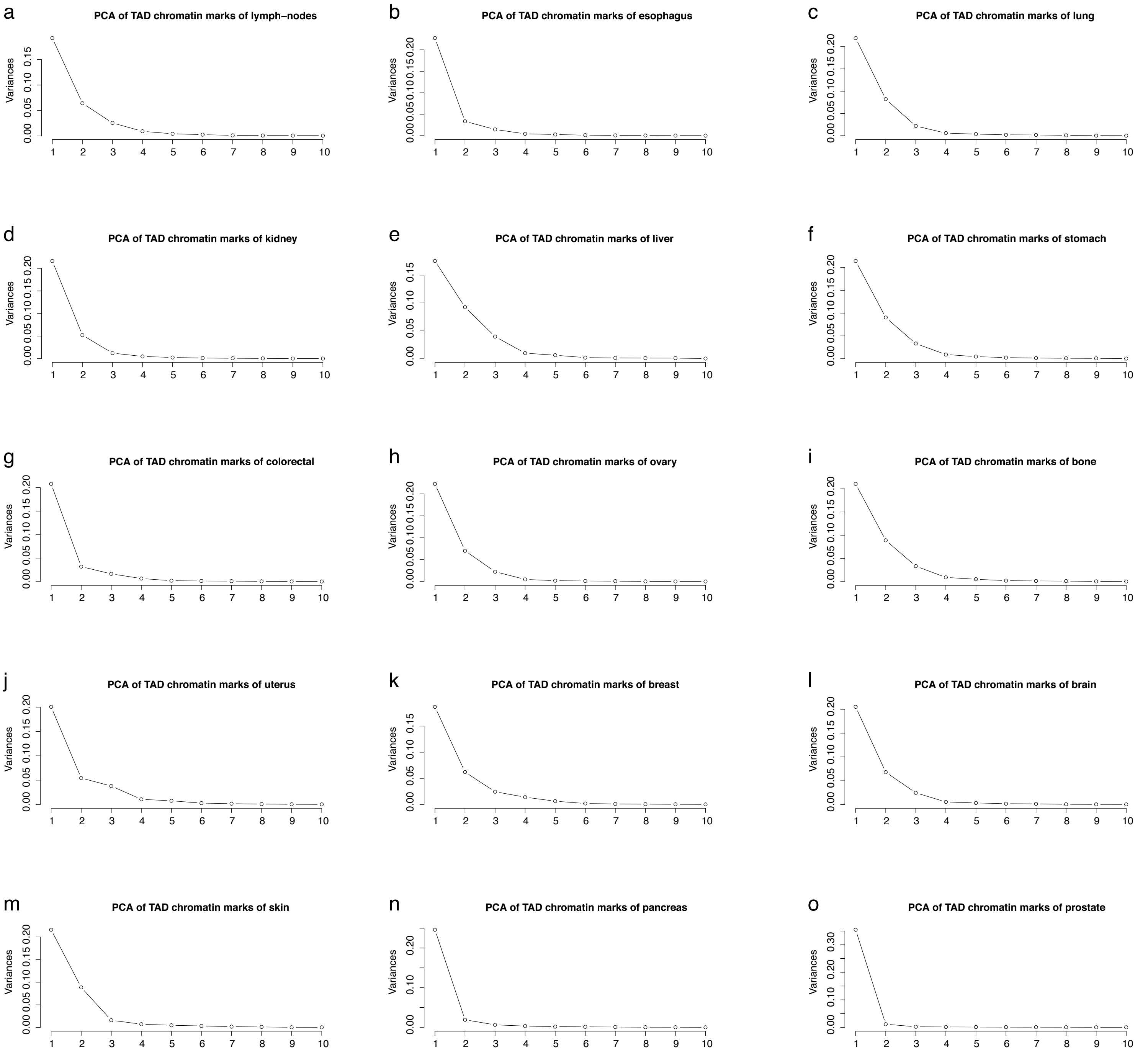

Contribution of the principal components of the chromatin marks enrichment to the TAD segments clustering.
Plots that show for each cancer type panel (a-o), the set of principal components that can explain the variance in the clustering of TAD segments based on their enrichment in the different chromatin marks. The maximum number of principal components that contribute to the clustering of TAD segments is 3 for each cohort in the study.

Supplementary Figure 11

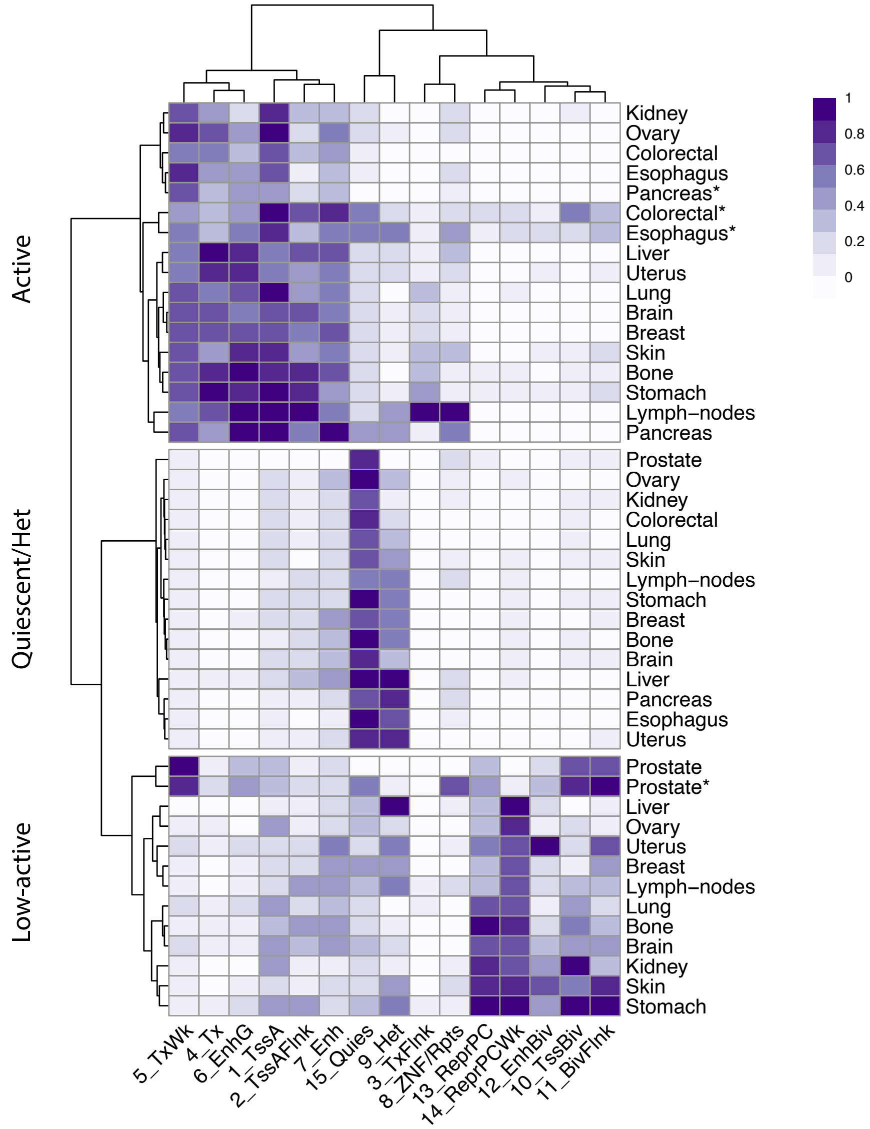

The three different classes of TAD segments across cancer types.
Heatmap that shows the concordance across the different cohorts in the three different classes of TAD segments (active, low active and quiescent). The scale goes from light to dark purple in ascending enrichment. The rows are clustered and split into three blocks. The top block is enriched in Active marks that include (TssA, Tx, EnhG, Enh), the middle block is enriched in Quiescent and Heterochromatin marks and the block in the bottom is enriched in Low-active and Repressed marks.

Supplementary Figure 12

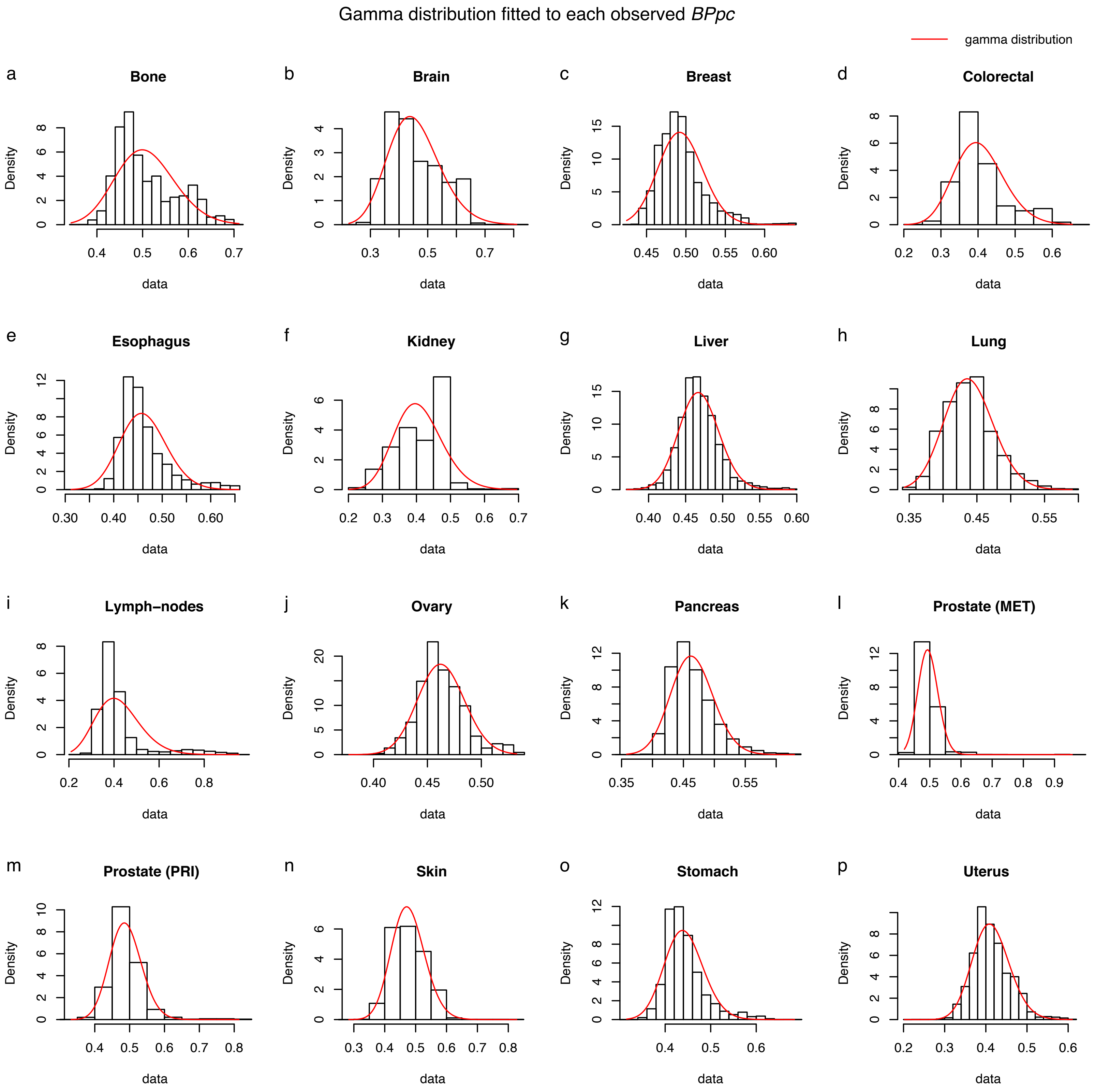

Gamma distribution of the observed BPpc.
Plot of the gamma distribution fitting to the distribution of the observed BPpc for each cohort panel (a-p). Therefore, we assume a gamma distribution for link function of the model.
