# Supplementary material for: Modeling tissue-specific breakpoint proximity of structural variations from whole-genomes to identify cancer drivers
Source: Nat Commun. 2022 Sep 26;13:5640. doi: 10.1038/s41467-022-32945-2 (PMC9512825; doi:10.1038/s41467-022-32945-2)
Supplement: Supplementary file 3 — Description to Additional Supplementary Information [file 41467_2022_32945_MOESM3_ESM.pdf]

List of supplementary Data

Supplementary Data 1: Dataset of SV calls for each cohort used in the study.

Supplementary Data 2: Genomic annotations and covariates used in the study and the source as well as the corresponding tissue type for the tissue-specific analysis.

Supplementary Data 3: Variability in the total number of samples, the explained deviance of the model and the variability in the cancer cell type composition for each cohort used in the study.

Supplementary Data 4: Results for the significantly recurrent rearrangement peaks. For each peak it annotates the chrom, the peak.start and peak.end, the peak.ID, the peak range (range_length), the number of breakpoints in the peak (# BP) , the number of samples in the peak (# sample), the number of SV in the peak (# SV), the peak area (peak.area), the peak recurrence score (PRs), the p-value for the peak recurrence score (PRs.pvalue), the FDR for the peak recurrence score (PRs.qvalue.BH) and cancer type (cancer_type).

Supplementary Data 5: Comparison of the overlap between the significantly recurrent rearrangement peaks detected by CSVDriver and the regions from PCAWG prediction using the same data. For each CSVDriver peak, it annotates the chromosome location (chromosome, start, end), peak id (peak.ID), cancer type (CancerType) and the number of cancer types that have peaks in the region (# of CancerType). The regions from PCAWG show the information reported in that study.

Supplementary Data 6: SV type composition of each significant peak for each cohort. The table shows the heterogeneity observed in the SV types within the peaks and marks the regions significantly enriched in one particular SV type compared to the pooled SV proportion.

Supplementary Data 7: Driver candidates predicted by CSVDriver. For each driver candidate, it annotates the cancer type (cancer_type), the peak ID (peak.ID), the peak location (chrom, peak.start, peak.end), the peak range (range_length), the number of breakpoints in the peak (# BP) , the number of samples in the peak (# sample), the number of SV in the peak (# SV), the peak area (peak.area), the peak recurrence score (PRs), the pvalue (PRs.pvalue) and the FDR (PRs.qvalue.BH) for the peak recurrence score using a test of fit for the Gamma distribution, the driver element (driver.elemet) that can be gene CDS, LncRNA and the enhancer ID, gene name (GeneName), element type (element_type), type of impact (type_of_impact) that can be SV_overlap or BP_disruption, number of SVs impacting the elemnet (#SV_GENE), number of samples with the impacted elemet (#sample_GENE) and the element rearrangement score (ERs), the asterisks in the last column mark for each peak most likely candidate reported in this study.

Supplementary Data 8: Summary table for cancer types with RNA-seq data. It includes for each cancer type the number of donors and the type of information that is available that can be (donors w/ SVs + Expr) Or (Donors w/ SVs).

SupplementaryData 9: Results for the significant peaks for single-sample rearrangements. For each peak we show the chrom (chromosome), the peak.start and peak.end, the peak.ID, the peak range (range_length), the number of breakpoints in the peak (# BP) , the number of samples in the peak (# sample), the number of SVs in the peak (# SV), the peak single-sample rearrangement score (SSRs), the

p-value (SSRs.pvalue) and the FDR (false discovery rate) for the peak single-sample rearrangement score (SSRs.qvalue.BH) using a test of fit for the Gamma distribution, and cancer type (cancer_type).

Supplementary Data 10: Comparison of the method's performance using GAM model from an independent cohort of the same cancer type. We used structural variation calls from three different ICGC cohorts (breast, prostate, and skin cancer), each of which are independent from the PCAWG datasets used to develop the method. (a) the left-side table shows the ICGC cohort top significant peaks (p-value < 0.025) output by adjusting the BPpc with the corresponding new computed model. (a) The right-side table shows the ICGC top significant peaks (p-value < 0.025) output by adjusting the BPpc with the previously computed model on PCAWG cohorts of the same cancer type. The p-values are the empirical probability of peak recurrence score using a test of fit for the Gamma distribution. (b) Table that shows the effect of cohort sample size on the explained variance.

Supplementary Data 11: The number and frequency of mutations for the mutated DNA repair genes in breast cancer. Equation of the extended GAM that includes the mutational status of DNA-repair genes as covariate.
