# Supplementary material for: Modeling tissue-specific breakpoint proximity of structural variations from whole-genomes to identify cancer drivers
Source: Nat Commun. 2022 Sep 26;13:5640. doi: 10.1038/s41467-022-32945-2 (PMC9512825; doi:10.1038/s41467-022-32945-2)
Supplement: Supplementary file 5 — Reporting Summary [file 41467_2022_32945_MOESM5_ESM.pdf]

nature portfolio

Corresponding author(s): Ekta Khurana
Alexander Martinez-Fundichely

Last updated by author(s): Aug 19, 2022



# Reporting Summary

Nature Portfolio wishes to improve the reproducibility of the work that we publish. This form provides structure for consistency and transparency in reporting. For further information on Nature Portfolio policies, see our Editorial Policies and the Editorial Policy Checklist.

## Statistics

For all statistical analyses, confirm that the following items are present in the figure legend, table legend, main text, or Methods section.

| n/a | Confirmed | |
|---|---|---|
| ☐ | ☒ | The exact sample size ($n$) for each experimental group/condition, given as a discrete number and unit of measurement |
| ☐ | ☒ | A statement on whether measurements were taken from distinct samples or whether the same sample was measured repeatedly |
| ☐ | ☒ | The statistical test(s) used AND whether they are one- or two-sided<br>*Only common tests should be described solely by name; describe more complex techniques in the Methods section.* |
| ☐ | ☒ | A description of all covariates tested |
| ☐ | ☒ | A description of any assumptions or corrections, such as tests of normality and adjustment for multiple comparisons |
| ☐ | ☒ | A full description of the statistical parameters including central tendency (e.g. means) or other basic estimates (e.g. regression coefficient) AND variation (e.g. standard deviation) or associated estimates of uncertainty (e.g. confidence intervals) |
| ☐ | ☒ | For null hypothesis testing, the test statistic (e.g. $F$, $t$, $r$) with confidence intervals, effect sizes, degrees of freedom and $P$ value noted<br>*Give P values as exact values whenever suitable.* |
| ☒ | ☐ | For Bayesian analysis, information on the choice of priors and Markov chain Monte Carlo settings |
| ☒ | ☐ | For hierarchical and complex designs, identification of the appropriate level for tests and full reporting of outcomes |
| ☒ | ☐ | Estimates of effect sizes (e.g. Cohen's $d$, Pearson's $r$), indicating how they were calculated |

*Our web collection on statistics for biologists contains articles on many of the points above.*

## Software and code

Policy information about availability of computer code

| | |
|---|---|
| Data collection | No software was used. The structural variation data was optioned form the PCAWG project |
| Data analysis | - The algorithm of the method have been develop in R script R version 3.6.2 (2019-12-12). The code is available at https://github.com/khuranalab/CSVDriver<br>The method use<br>- R package 'mgcv' version 1.8-28 for fitting the GAM.<br>- R Package 'fitdistrplus' version 1.1-1 for fitting the gamma distribution theoretical density.<br>- R Package 'biovizBase' version 1.44.0 function 'GCcontent' for computing the GC content<br>- R Package 'kpPlotDensity' version 1.10.0 function kpPlotDensity for computing the gene density |

For manuscripts utilizing custom algorithms or software that are central to the research but not yet described in published literature, software must be made available to editors and reviewers. We strongly encourage code deposition in a community repository (e.g. GitHub). See the Nature Portfolio guidelines for submitting code & software for further information.

## Data

Policy information about availability of data

All manuscripts must include a data availability statement. This statement should provide the following information, where applicable:

- Accession codes, unique identifiers, or web links for publicly available datasets
- A description of any restrictions on data availability
- For clinical datasets or third party data, please ensure that the statement adheres to our policy

All the data generated in this study are provided in the Supplementary Information/Source Data file.
The input SV data used in the study is described in Supplementary Table 1 and are available for download at https://dcc.icgc.org/releases/PCAWG. In accordance with the data access policies of the ICGC and TCGA projects, accessing potentially identifying information need authorisation granted by applying to the TCGA Data Access Committee (DAC) via dbGaP (https://dbgap.ncbi.nlm.nih.gov), and to the ICGC Data Access Compliance Office (DACO; http://icgc.org/daco). The input SV data for metastatic prostate cancer were generated and is available in the supplementary material at Quigley, D.A., et al. (PMID: 30033370) and Viswanathan, S.R., et al. (PMID: 29909985)
The covariates input data is provided at the CSVDriver's GitHub repository within the publishing release asset list (https://github.com/khuranalab/CSVDriver/releases/tag/v0.1.0-beta.1) corresponding to DOI: 10.5281/zenodo.6969761, and the description is provided in Supplementary Table 2 it includes
- The input tissue-specific chromatin state marks are available for download at Roadmap Epigenomics Mapping Consortium portal (https://egg2.wustl.edu/roadmap/web_portal/).
- The RT input datasets are available for download at ENCODE portal (https://www.encodeproject.org/).
- The FS annotation input data is available for download at the HumCFS database (https://webs.iiitd.edu.in/raghava/humcfs/).
- The repClass input data from repeat masker is available for download at the UCSC genome browser (https://hgdownload.soe.ucsc.edu/).
- The input annotation of genome LADs is available in the supplementary material at Akdemir, K. C. et al. (PMID: 32024999)
- The input TADs annotations is available at the 3D Genome Browser (http://3dgenome.fsm.northwestern.edu/index.html).
- The information for verifying cancer related genes is available at CancerMine database (http://bionlp.bcgsc.ca/cancermine/) and COSMIC portal (https://cancer.sanger.ac.uk/cosmic).

## Human research participants

Policy information about studies involving human research participants and Sex and Gender in Research.

| | |
|---|---|
| Reporting on sex and gender | No sex and gender analysis was performed. It was no needed under the scope of this study. |
| Population characteristics | No population characteristics were used in this method. It was not necessary under the scope of this study. |
| Recruitment | No participants were recruited for this analysis. It was not necessary under the scope of this study. |
| Ethics oversight | It was not necessary under the scope of this study. |

Note that full information on the approval of the study protocol must also be provided in the manuscript.

# Field-specific reporting

Please select the one below that is the best fit for your research. If you are not sure, read the appropriate sections before making your selection.

☒ Life sciences ☐ Behavioural & social sciences ☐ Ecological, evolutionary & environmental sciences

For a reference copy of the document with all sections, see nature.com/documents/nr-reporting-summary-flat.pdf

# Life sciences study design

All studies must disclose on these points even when the disclosure is negative.

| | |
|---|---|
| Sample size | We applied the method using the total number of sample available for each cancer cohort. Then for each cohort we perform a sample-size power calculations for detecting significant peaks of breakpoint clustering. We confirm that the sample size currently available for most cohorts provides 90% power of detection for peaks with a prevalence of 25% or more. |
| Data exclusions | Cancer cohort that present low burden of SV where exclude form the analysis because that imply that these cancer are less like to be driven by SV rearrangement. |
| Replication | The method was applied separately to each cohort of different cancer types and demonstrates the validity of the tissue-specific approach. Furthermore, the result in the same cancer type was positively replicated using independent cohorts for prostate, breast and skin cancer. |
| Randomization | No randomization was performed. It was no needed under the scope of this study. The factor that affect the break point proximity were controlled by applying a GAM model. It uses the corresponding genomics covariates to assess the the expected break point proximity per genome location. |

Blinding

There is no observer bias because there are no a priori expectations about genome-wide clustering of breakpoints. Therefore no blinding was performed. It was not necessary under the scope of learn from the data of this study.

# Reporting for specific materials, systems and methods

We require information from authors about some types of materials, experimental systems and methods used in many studies. Here, indicate whether each material, system or method listed is relevant to your study. If you are not sure if a list item applies to your research, read the appropriate section before selecting a response.

## Materials & experimental systems

| n/a | Involved in the study |
|---|---|
| ☒ | ☐ Antibodies |
| ☒ | ☐ Eukaryotic cell lines |
| ☒ | ☐ Palaeontology and archaeology |
| ☒ | ☐ Animals and other organisms |
| ☒ | ☐ Clinical data |
| ☒ | ☐ Dual use research of concern |

## Methods

| n/a | Involved in the study |
|---|---|
| ☒ | ☐ ChIP-seq |
| ☒ | ☐ Flow cytometry |
| ☒ | ☐ MRI-based neuroimaging |
